# Anaerobic demethylation of guaiacyl-derived monolignols enabled by a designed artificial cobalamin methyltransferase fusion enzyme†

**DOI:** 10.1039/d2ra08005b

**Published:** 2023-02-15

**Authors:** Christopher Grimm, Simona Pompei, Kristina Egger, Michael Fuchs, Wolfgang Kroutil

**Affiliations:** a Institute of Chemistry, University of Graz, NAWI Graz Heinrichstraße 28 8010 Graz Austria wolfgang.kroutil@uni-graz.at; b BioTechMed Graz 8010 Graz Austria; c Field of Excellence BioHealth, University of Graz 8010 Graz Austria

## Abstract

Lignin-derived aryl methyl ethers (*e.g.* coniferyl alcohol, ferulic acid) are expected to be a future carbon source for chemistry. The well-known P450 dependent biocatalytic *O*-demethylation of these aryl methyl ethers is prone to side product formation especially for the oxidation sensitive catechol products which get easily oxidized in the presence of O_2_. Alternatively, biocatalytic demethylation using cobalamin dependent enzymes may be used under anaerobic conditions, whereby two proteins, namely a methyltransferase and a carrier protein are required. To make this approach applicable for preparative transformations, fusion proteins were designed connecting the cobalamin-dependent methyltransferase (MT) with the corrinoid-binding protein (CP) from *Desulfitobacterium hafniense* by variable glycine linkers. From the proteins created, the fusion enzyme MT-L5-CP with the shortest linker performed best of all fusion enzymes investigated showing comparable and, in some aspects, even better performance than the separated proteins. The fusion enzymes provided several advantages like that the cobalamin cofactor loading step required originally for the CP could be skipped enabling a significantly simpler protocol. Consequently, the biocatalytic demethylation was performed using Schlenk conditions allowing the *O*-demethylation *e.g.* of the monolignol coniferyl alcohol on a 25 mL scale leading to 75% conversion. The fusion enzyme represents a promising starting point to be evolved for alternative demethylation reactions to diversify natural products and to valorize lignin.

## Abstract 2

Lignin-derived aryl methyl ethers (*e.g.* coniferyl alcohol, ferulic acid) are demethylated under anaerobic conditions using a cobalamin dependent fusion enzyme, which combines the cobalamin carrier protein and the methyltransferase.

## Graphical abstract

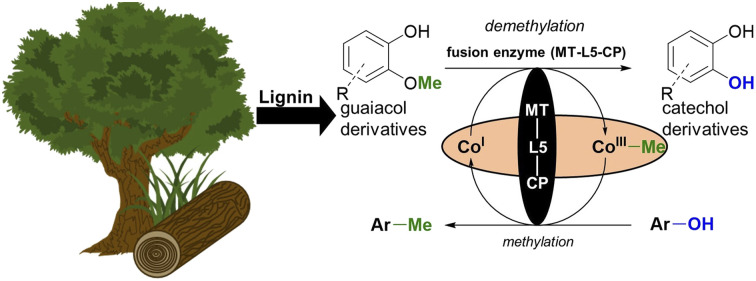

## Introduction

With annual quantities of 140 million tons,^1^ lignin is the most abundant natural polymer with aromatic moieties^2,3^ and the second most abundant substance of plant biomass on Earth after cellulose.^4–8^ Currently a huge amount of this highly branched and complex polymer is mainly combusted to generate heat and power during pulping and biofuel production.^7,9–14^ As a consequence of the need to use renewables as carbon sources, lignin is considered as a sustainable alternative for phenolic oil-based chemicals.^15–17^ Until now, various chemical methodologies are known to degrade lignin^18,19^ such as the pyrolysis (thermolysis),^20–23^ hydrolysis,^24,25^ depolymerization using chemical functionalization,^26,27^ oxidation^28,29^ or reduction.^30,31^ To expand the transformations of lignin in biorefining processes,^32^*O*-demethylation is of interest. The most common chemical protocols for demethylation require toxic reagents or harsh reaction conditions (*e.g.* hydrogen bromide and boron tribromide;^33–36^ for methylation methyl iodide and dimethyl sulfate are commonly applied^37–39^). For a possible sustainable alternative, enzymes may be considered working under mild and aqueous conditions.^40,41^

**Scheme 1:**
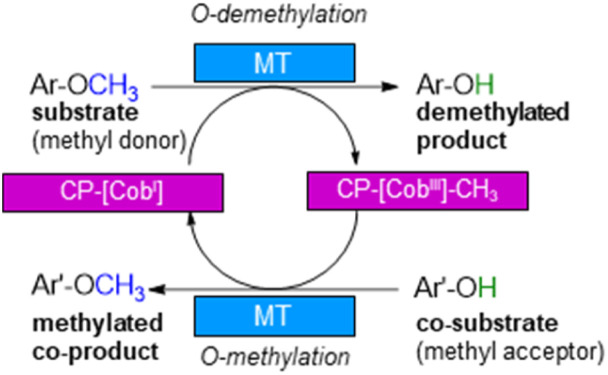
Cobalamin-dependent demethylation with a sacrificial acceptor requiring a methyltransferase (MT) and the carrier protein (CP).

For demethylation oxidative enzymes^42–50^ have been mainly used while tetrahydrofolate^51,52^ dependent or SAM-dependent methyltransferases^53–56^ have been used for *O*-methylation. In contrast, cobalamin-dependent methyltransferases originating *e.g.* from strictly anaerobic organisms such as *Methanosarcina barkeri*, *Acetobacterium dehalogenans*, *Desulfitobacterium hafniense*, *Moorella thermoaceticum* and *Methylobacterium* sp.^57–59^ catalyze both reactions, the methylation of phenols and the demethylation of methyl phenyl ethers in a reversible manner (Scheme 1).^60–64^ Depending on the methyl acceptor used, irreversible demethylation has been shown.^65^

It is worth to note, that in contrast to oxidative demethylating enzymes,^42–50^ cobalamin-dependent methyltransferases do not require molecular oxygen for demethylation. This is important, as molecular oxygen may initiate undesired side reactions of the phenolic substrates/products such as polymerisation especially for catechol derivatives.^66–68^

The cobalamin-dependent (de)methylation requires a methyltransferase (MT) and a corrinoid-binding protein (CP).^60,61^ Thus actually two proteins are needed for this transformation. This implies several challenges such as (i) separate expression of the MT and the CP, as well as (ii) an excess of the CP compared to the MT for optimal procedure, probably due to a low binding constant of CP to MT, (iii) a time and reagent-intensive loading step of methylcobalamin onto the CP (incubation for >2 h at 4 °C followed by a desalting step) and (iv) the setup of the reaction requires a glovebox to ensure an oxygen free environment, since cobalamin is sensitive to molecular oxygen in oxidation state one. To address the first two issues, the design of a fusion enzyme^69–74^ linking the two proteins MT and CP was envisioned. Identifying the best suitable fusion protein is still an empirical process, which has to be done by trial and error. In some cases, fusion enzymes have been reported to be superior in comparison to their separated parts either due to improved protein expression^69,70^ or improved stability.^71–74^ Having a fusion protein in hand for the cobalamin dependent methyltransfer would especially ease further enzyme engineering as then a single protein can be optimized instead of two proteins. With such a fusion enzyme all other issues were successfully addressed in this study.

## Results and discussion

### Design of fusion enzymes

**Fig. 1:**
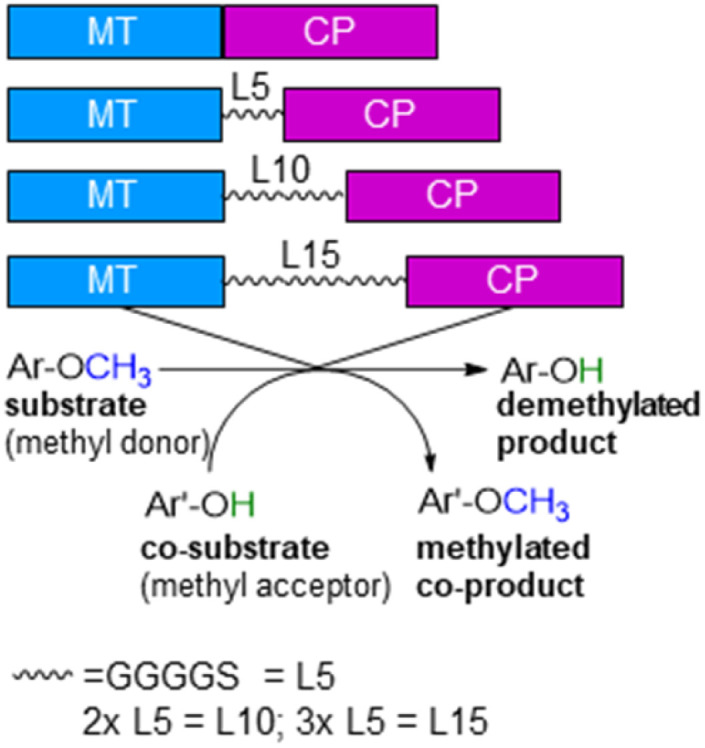
Design of recombinant fusion enzymes for *O*-demethylation.

To develop cobalamin-dependent methyltransfer reactions as a general tool, a single protein would be desired instead of the two proteins (MT and CP) as well as an easily applicable procedure. To address the first point, the design of a fusion protein linking MT and CP was envisioned. For this purpose the cobalamin-dependent methyltransferase (*dhaf*-MT, 37.5 kDa, 327 amino acids, expression level 3.5%) and the corrinoid-binding protein (*dhaf*-CP, 21.4 kDa, 212 amino acids, expression level 3%) originating from the strictly anaerobic bacterium *Desulfitobacterium hafniense* were investigated.^60–63^ To decide whether the MT is at the N-terminus or at the C-terminus, *dhaf*-MT and *dhaf*-CP were aligned to the natural methyltransfer protein cmuA (c̲hlorom̲ethane u̲tilisation)^75,76^ from *Hyphomicrobium chloromethanicum*, that contains a methyltransfer domain and a cobalamin domain*.*^77^ It is worth to note that this enzyme cmuA has never been expressed in *E. coli* before and also all our trials to express cmuA failed. In the alignment *dhaf*-MT fitted best for the N-terminus and *dhaf*-CP for the C-terminus (21%, EMBOSS needle, ESI†). Subsequently, four designs of fusion enzymes were constructed linking MT and CP (Fig. 1) using either no linker (MT-CP, 58.9 kDa, 539 amino acids) or linkers of varied length. Thereby, flexible linkers with glycine-rich sequences (GGGGS)_*n*_^*7*8,79^ were introduced. The length of the linker incorporated between the MT and CP was varied taking *n* as 1 to 3 (*n* = 1: MT-L5-CP, 59.8 kDa, 548 amino acids; *n* = 2: MT-L10-CP, 60.1 kDa, 553 amino acids; *n* = 3: MT-L15-CP, 60.4 kDa, 558 amino acids). The linkers should allow more flexibility between the two protein entities (MT-CP) and also reduce the chance to form inclusion bodies.^80^ All recombinant fusion enzymes were successfully expressed in soluble form in *E. coli* BL21(DE3) showing the appropriate bands around 60 kDa [Fig. S10,† with expression levels 6.8% (MT-CP), 4.9% (MT-L5-CP), 4.7% (MT-L10-CP), 3.9% (MT-L15-CP)].

**Scheme 2:**
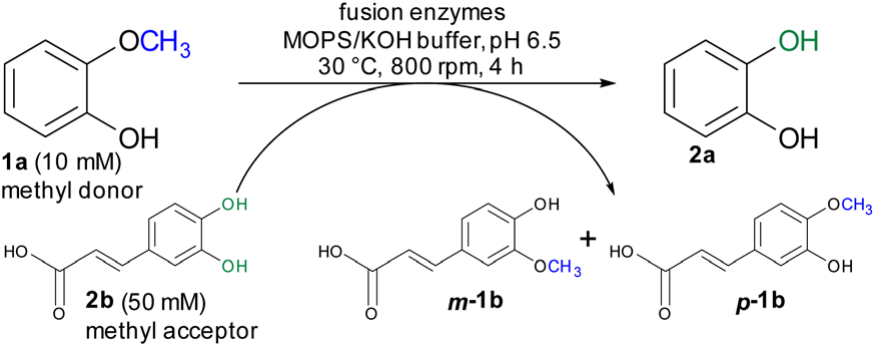
Demethylation of guaiacol 1a as model reaction using caffeic acid 2b as methyl acceptor.

**Fig. 2:**
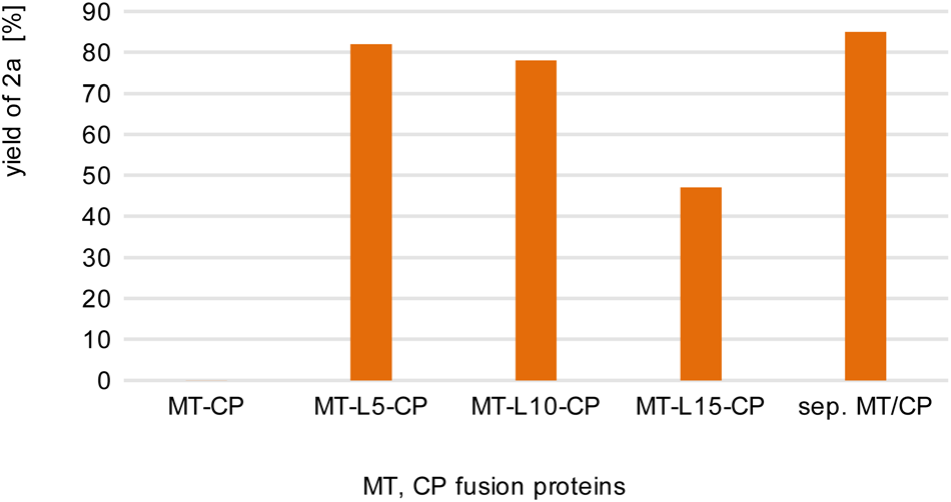
Comparison of fusion enzymes with the separated MT/CP. Reaction conditions: 1a (10 mM), 2b (50 mM), MT-X-CP [as cell-free extract (CFE), corresponding to 0.6 mM pure MT-L5-CP] or separated MT + 1.5xCP (corresponding to 1 mM pure *dhaf*-CP) in MOPS buffer (50 mM, pH 6.5, 150 mM KCl) at 30 °C, 800 rpm in a glovebox (N_2_) for 4 h. Product formation was analyzed *via* calibration curves of the corresponding reference compounds on HPLC-UV (for complete data set see Table S1†).

For initial biocatalytic test reactions, the recombinant fusion enzymes were loaded with the cofactor methylcobalamin (MeCob) in a separate operating step as previously described.^60,61^ Testing the fusion enzymes with the model substrates guaiacol 1a (methyl donor) and caffeic acid 2b as methyl acceptor (Scheme 2), all fusion enzymes except the one without any linker (MT-CP) led to conversion (Fig. 2).

This may indicate that a certain distance/flexibility between the MT-part and the CP-part is required to enable the reaction. Regarding the length of the linkers, a clear trend was observed: the enzyme with the shortest linker MT-L5-CP gave best results allowing to demethylate 82% of guaiacol 1a. The result obtained with this fusion protein MT-L5-CP was comparable to the optimized experiment using the non-linked MT and CP at a ratio of 1 : 1.5, thus the linked-catalyst led to similar conversion as the optimized reaction with the non-linked catalyst but having now the two parts at a 1 : 1 ratio. This can already be seen as a significant advancement, since in the fusion enzyme the ratio of the CP domain and the MT part is obviously 1 : 1, while for optimal performance of the separated proteins, the CP had to be present in at least 150% compared to the MT.

### Direct addition of MeCob and operational window of reaction parameters

In the reaction protocol used above an extra loading step of the CP with cobalamin was performed involving buffer exchange with PD columns as previously reported for reactions with separated CP and MT.^81^ We wondered whether for the fused protein these extra operational steps can be avoided by adding MeCob directly to the fused protein. Furthermore, the operational windows for the reaction parameters of the best fusion enzyme MT-L5-CP were evaluated regarding (i) the MeCob concentration, (ii) the ratio between methyl donor and acceptor, (iii) protein concentrations and (iv) reaction times.

First, varied concentrations of MeCob (7.4 μM-2.2 mM) were tested and added directly to the MT-L5-CP fusion protein and subsequently the demethylation of guaiacol 1a was assayed (Fig. S1†). The results showed that indeed the fusion protein enabled a shorter operative protocol by circumventing the separate cobalamin loading steps by direct addition. Best results were obtained by adding MeCob at a concentration of 0.37 mM.

The ratio between methyl donor and acceptor is of interest because the reaction can be pushed either toward demethylation or methylation by using an excess of methyl acceptor or donor, respectively. When the methyl donor guaiacol 1a was applied in a two-fold or three-fold molar excess, more methylated products (up to 74% *m*-1b, *p*-1b) were formed than by using an equimolar ratio (42% conv. 2a, 57% conv. *m*-1b, *p*-1b, Fig. S2†). On the other hand, a three-fold molar excess of the methyl acceptor caffeic acid 2b showed best results leading to 68% demethylation of guaiacol 1a. In comparison to the reaction using non-fused proteins, the fusion enzyme MT-L5-CP reached higher conversion faster requiring at lower methyl acceptor concentration (three-fold molar excess) compared to the five-fold molar excess of methyl acceptor for the separated proteins.^60,61^

Investigating the amount of catalyst employed, it turned out that by lowering the enzyme concentration provided as cell-free extract (CFE) from 0.6 to 0.18 mM, even higher product formations were achieved at the conditions employed (85% 2a, squares, Fig. S3, Table S2†). At this concentration of the fusion enzyme (corresponding to 0.18 mM pure MT-L5-CP), the ratio between enzyme and the experimentally determined optimum amount of MeCob (0.37 mM) corresponded to a 2-fold equimolar excess of the cofactor.

Using purified enzyme MT-L5-CP (1.3 mM), the model substrate guaiacol 1a was demethylated to catechol (51% 2a, square, Fig. S4, Table S3†) at the conditions employed above after 4 h. Comparing the product formation using comparable amounts of CFE and purified enzyme, it turned out that the CFE preparation is about 200 times faster than using purified MT-L5-CP (Tables S2 and S3,† at 0.17 mM enzyme concentration). Hence, the use of CFE is advantageous, probably due to a stabilizing effect by other proteins present in the CFE.^82,83^ Following the time course of the reaction using CFE of MT-L5-CP, the plateau of product formation was reached after 3 h (Fig. S5†).

### *O*-Demethylation of selected substrates

Selected substrates (1a, *m*-1b, 1c-d) were investigated for being demethylated employing the fusion enzyme MT-L5-CP *via* methyl transfer using various methyl acceptors (2a-b, 2e-f, Table 1). Highest conversions were achieved with 2-methoxy-5-methylphenol 1c in the presence of the methyl acceptors 3,4-dihydroxybenzyl alcohol 2f (entry 7) and catechol 2a (entry 8) leading to 96% and 94% conversion, respectively (Table S4† for detailed information). Furthermore, three lignin related molecules namely ferulic acid *m*-1b, coniferyl alcohol 1d and sinapyl alcohol 1g were investigated. Ferulic acid *m*-1b was demethylated with up to 68% conversion (entries 4–5), while sinapyl alcohol was not transformed.

**Table 1:** Substrate scope of MT-L5-CP for the *O*-demethylation of guaiacol derivatives

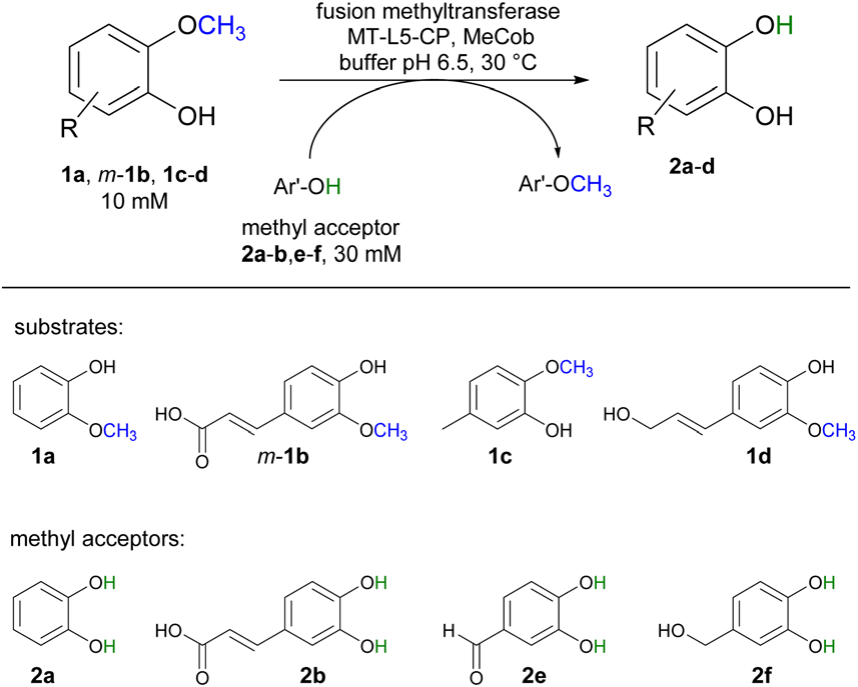			
Entry	Substrate	Co-substrate	Demethylated product 2^a^ [%]
1	1a	2b	86 ± 6
2	1a	2e	77 ± 2
3	1a	2f	85 ± 1
4	*m*-1b	2a	68 ± 1
5	*m*-1b	2f	67 ± 3
6	1c	2e	86 ± 5
7	1c	2f	96 ± 2
8	1c	2a	94 ± 3
9	1d	2b	68 ± 1
10	1d	2a	83 ± 2

a Reaction conditions: substrate 1a, *m*-1b, 1c-d (10 mM), co-substrate 2a-b, 2e-f (30 mM), MT-L5-CP (CFE corresponding to 0.18 mM pure MT-L5-CP) in MOPS buffer (50 mM, pH 6.5, 150 mM KCl) with MeCob (0.5 mg mL^−1^, 0.37 mM) at 30 °C, 800 rpm in Eppendorf Thermomixer (1.5 mL) for 3 h under inert atmosphere (glovebox). Total volume: 120 μL. The conversions were determined on HPLC-UV using calibration curves.

In addition, coniferyl alcohol 1d was efficiently demethylated to caffeoyl alcohol 2d with either 2b (68% conv., entry 9) or catechol 2a as acceptor molecule (83% conv., entry 10). The obtained product caffeoyl alcohol 2d is a forty times more valuable product compared to coniferyl alcohol (398 €/10 mg 2d compared to 9.7 €/10 mg 1d; Sigma Aldrich). Synthetic routes to caffeoyl alcohol 2d have been reported previously either chemically from ethyl 3,4-dihydroxycinnamate (81% yield, LiAlH_4_/BnCl)^84^ and caffeic acid 2b (two steps, 60% yield, MeOH, H_2_SO_4_, LiAlH_4_, AlCl_3_)^85^ or by using *E. coli* from caffeic acid 2b (0.03 mM 2d compared to our approach with 8.3 mM 2d).^86^ The biocatalytic cobalamin dependent demethylation of these compounds leading to products with an oxidation sensitive 1,2 aromatic diol is only feasible because the cobalamin dependent methyl transferase does not require any oxidant like molecular oxygen as needed by P450 demethylating enzymes.^42–50^

### Comparison of activity of MT-L5-CP and separated MT/CP

To compare the activity of the fusion protein and the separated MT/CP enzyme as CFE preparation, protein concentrations were determined on SDS-PAGE (Fig. S6†) using densitometry [(MT-L5-CP 9.2 pmol), *dhaf*-MT (12.6 pmol) and *dhaf*-CP (19.3 pmol)] and normalized to the amount of MT-L5-CP for an equal comparison between all biocatalysts. The demethylation of 1c (10 mM methyl donor) was followed in the presence of 2f and MeCob (0.37 mM) over 5–20 min (MT-L5-CP: Fig. S7, MT/CP separated: Fig. S8†). Control experiments showed that the *E. coli* host itself does not possess any enzymes catalyzing this reaction. From the initial phase the activity could be deduced to be 10 mU per mg of pure MT-L5-CP (1 U corresponds to the μmol of 1c transformed per min at the above given conditions) which was comparable to the activity determined for the non-linked proteins MT/CP (9.8 mU mg^−1^) at their optimized conditions. Thus, the linkage between MT and CP allowed to maintain the activity while getting now access to a single protein bearing all required features and which can be expected to be now evolvable to reach even higher activity.

### Demethylation using Schlenk technique (25 mL scale)

**Scheme 3:**
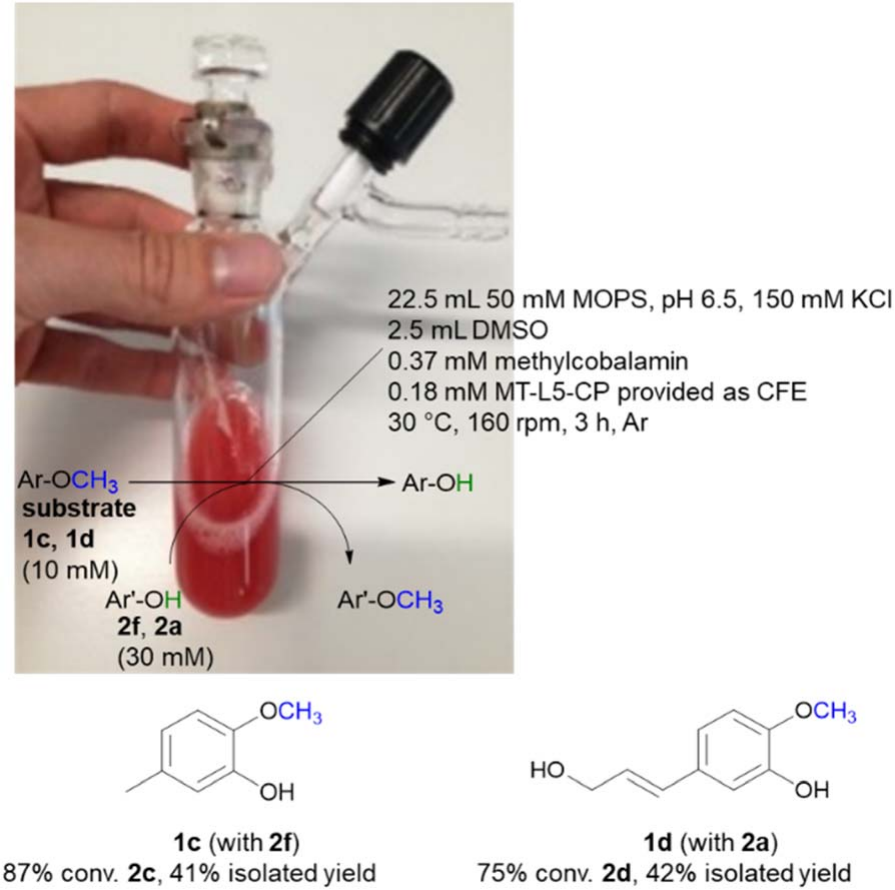
Semi-preparative demethylation of 1c and 1d catalyzed by MT-L5-CP using Schlenk technique.

While the experiments described above were conducted in a glovebox specific for biocatalysis (non-moisture sensitive O_2_-sensor), the now single protein catalyst was envisioned to allow a procedure using Schlenk equipment. For this purpose, the substrate 2-methoxy-5-methylphenol 1c was demethylated on a 25 mL semi preparative scale using 2f as methyl acceptor. Remarkably, the 25 mL biotransformation with the fusion enzyme MT-L5-CP led to the demethylated product with 87% conv. (Scheme 3, Fig. S9† for detailed information), thus it was comparable to the biotransformation on analytical scale (120 μL, 96% conv. 1e, Table 1, entry 7).

Subsequently the demethylation of the lignin related coniferyl alcohol 1d was performed on a 25 mL scale, simulating a possible valorization of lignin derived aromatics. The fusion protein MT-L5-CP catalyzed the demethylation of coniferyl alcohol 1d to caffeoyl alcohol 2d with 75% conversion. The corresponding products 2c and 2d were isolated with 41–42% isolated yield and confirmed by ^1^H and ^13^C NMR (2a, Fig. S20, S21; 1a, S22, S23; 1d, S24–S27; 2d, S28, S29†).

## Conclusions

Self-sufficient fusion enzymes were successfully designed for the biocatalytic cobalamin dependent *O*-demethylation of lignin related compounds. The fusion enzymes investigated comprise the N-terminal methyltransferase (*dhaf*-MT) and the C-terminal corrinoid-binding protein (*dhaf*-CP) both from *D. hafniense*, which were connected by a flexible glycine linker (GGGGS)_*n*_,^79^ whereby a single (GGGGS)-unit turned out to be suited best (MT-L5-CP). The availability of a fusion protein containing the cobalamin carrier domain and the methyl transfer domain represents a significant advancement for this reaction as the fusion protein allowed now to circumvent the previously required preloading step of the carrier protein. The fusion system enabled also to successfully transform various substrates. For instance, the catalytic *O*-demethylation of 2-methoxy-5-methylphenol 1c reached 96% conversion using MT-L5-CP. Such high conversion *via* biocatalytic demethylation leading to these oxidation sensitive catechol derivatives is only possible using anaerobic conditions. The handling of the fusion protein allowed now also to perform the demethylation under inert atmosphere using a Schlenk line. Semi preparative demethylations of *e.g.* coniferyl alcohol was demonstrated on a 25 mL scale in an oxygen-free atmosphere reaching conversions between 75–85%. A clear advantage of having the MT and CP combined in the fusion enzyme is that only a single protein needs to be expressed, thereby saving one fermentation process. The fusion enzyme also represents also an ideal starting point for enzyme engineering to improve the binding of cobalamin and especially the activity even further.

## Experimental

### DNA sequences

All DNA sequences with and without glycine linkers (GGGGS)_*n*_^79^ were ordered from General Biosystems (see ESI†). The flexible linkers were incorporated between the restriction sites *XhoI* and *EcoRI* between these two proteins. All genes were cloned into the pET28a(+) vector (*NdeI* and *HindIII*) and transformed in *E. coli* BL21(DE3).

### Expression and purification

*Escherichia coli* BL21/Lemo21(DE3) was cultivated in lysogeny broth medium (0.5/1L LB) supplemented with kanamycin (50 μg mL^−1^) for the fusion enzymes or ampicillin (100 μg mL^−1^) for *dhaf*-MT and *dhaf*-CP. The inoculation was performed using 1% (v/v) from the over-night culture at 37 °C and 120 rpm. When cultures reached an optical density OD_600_ of 0.6–0.8, either 0.5 mM IPTG (fusion enzymes) or 0.2 μg mL^−1^ anhydrotetracycline (*dhaf*-MT and *dhaf*-CP) was added and the incubation continued for 24 h at 25 °C and 120 rpm. The cells were harvested by centrifugation (5000 rpm, 4 °C, 10 min). To prepare the lyophilized CFE, cells were resuspended in MOPS buffer (50 mM, pH 6.5, 150 mM KCl, 7 mL buffer to 1 g wet cells) and disrupted by ultrasonication on ice (40% amplitude, 3 × 6 min, pulse on 1 s, pulse off 2 s) using a Sonics & Materials Vibra Cell CV26 (13 mm tip, amplitude range 36–240). After the crude cell extract was separated from the cell debris by centrifugation (30 min, 14 000 rpm, 23 519 g), the extract was either frozen in liquid nitrogen and lyophilized over-night or further used for purification. The fusion enzyme MT-L5-CP was purified with ion-metal affinity chromatography on Ni-NTA, followed by size-exclusion chromatography on Superdex S75 16/60 according to the manual (Cytiva).

The CFE of all used proteins (Fig. S10†) and purified MT-L5-CP (Fig. S11†) were analyzed on SDS-PAGE and stored at 4 °C (−20 °C for pure sample) or instantly used for further experiments.

### Preparation of biocatalysts for the methyl transfer

All recombinant fusion enzymes and the *dhaf*-MT and *dhaf*-CP were used as freeze-dried cell free extracts (CFE) or purified preparations. Since the cofactor methylcobalamin (MeCob) is oxygen-sensitive, biocatalytic reactions were performed in triplicates in degassed buffers under inert atmosphere (99.8% N_2_, 5 bar) using a MBraun LABstar glove box equipped with a MB-OX-EC O_2_-sensor. Initial experiments were conducted with a prior MeCob loading step (chapter 3.5.1 *Holo*-CP and *holo*-fusion enzyme loading, ESI†) according to literature^60,61^ to ensure the active *holo*-form of fusion enzymes or *dhaf*-CP, because *E. coli* does not produce cobalamins.^87^ Model substrates guaiacol 1a (10 mM, methyl donor) and caffeic acid 2b (30 or 50 mM, methyl acceptor) were used in all experiments to analyze the operational window. All other biotransformations were performed under optimized reaction conditions as described below.

### Biocatalytic methyl transfer reactions

All biocatalytic reactions (120 μL analytical scale) were performed under inert atmosphere (99.8% N_2_, 5 bar) in a glove box. The lyophilized CFE of MT-L5-CP (60 mg mL^−1^ CFE corresponds to 27 mg mL^−1^, 0.45 mM pure MT-L5-CP) was dissolved in MOPS buffer (50 mM, pH 6.5, 150 mM KCl) with the direct addition of MeCob (0.5 mg mL^−1^, 0.37 mM). The reaction was started by adding the substrates (10 mM methyl donors 1a, *m*-1b, 1c or 1d) and co-substrates (30 mM methyl acceptors 2a, 2b, 2e or 2f) at 30 °C and 800 rpm in Eppendorf Thermomixer (1.5 mL) for 3 h. All substrate and co-substrate stocks (100 mM) were dissolved in MOPS buffer (50 mM, pH 6.5, 150 mM KCl) supplemented with 20% v/v DMSO (end-conc.: 10% v/v DMSO).

### Sample work-up for HPLC analysis

All reactions were quenched (10 vol%) by addition of MeCN (final conc. 60 vol%), mixed thoroughly and incubated for 20 min at room temperature. Afterwards, water (30 vol%, HPLC pure) was added and the denatured protein was removed by centrifugation (14 000 rpm, 15 800 rcf, 10 min). The supernatant was filtered through a pipette tip filled with cotton and the conversions were analyzed by HPLC-UV equipped with a Luna C18 column (mobile phase: water and MeCN with 0.1% trifluoroacetic acid, TFA, *Methods A* and *B*, chapter 4.2.1 Method for HPLC-UV and HPLC-MS, ESI†). Amount were deduced *via* calibration curves using reference compounds. All retention times and the corresponding *k*-values of both methyl donors and acceptors are summarized in Table S6 (ESI†).

### Semi-preparative biotransformation on Schlenk line

The MOPS buffer (50 mM, pH 6.5, 150 mM KCl) and DMSO were degassed (Ar) and aluminum foil was applied on the 50 mL Schlenk vessel due to the light and oxygen-sensitive cofactor. The vessel was flushed three times by alternating an argon stream and vacuum. For the biotransformation, the liquids were added first (22.5 mL MOPS buffer, 2.5 mL DMSO, 10 vol% final conc.) followed by the solid reagents including the lyophilized CFE of MT-L5-CP (1.5 g), MeCob (12.5 mg, 0.37 mM), the substrates 2-methoxy-5-methylphenol 1c (10 mM, 34.5 mg) or coniferyl alcohol 1d (10 mM, 52 mg) and the co-substrates 3,4-dihydroxybenzyl alcohol 2f (30 mM, 105 mg) or catechol 2a (30 mM, 82 mg). The Schlenk vessel was sealed with a glas stopper wrapped with parafilm and locked with a clamp. The 25 mL semi-preparative scale reaction was incubated at 30 °C and 160 rpm in an incubator shaker (Multitron Infors Ht) for 3 h.

After the reaction was completed, the sample was distributed in 1.5 mL Eppendorf tubes (500 μL) and extracted with EtOAc (3 × 500 μL) (otherwise at higher extraction volumes a gel-like precipitate formed). The combined organic fractions were dried (Na_2_SO_4_) and filtered through cotton filters. The crude was purified by column chromatography (1c and 2f: SiO_2_, EtOAc/cyclohexane 1 : 3 to 1 : 2 to 1 : 1, washing with 100% EtOAc at end) or automatically by the Biotage Selekt instrument (1d and 2a: SiO_2_, *ø* = 20 μm, 5 g, 1 CV = 9 mL; EtOAc/cyclohexane, 3 CV 10% EtOAc, 10 CV 10–40% EtOAc, 2 CV 40% EtOAc, 10 CV 40–70% EtOAc and 9 CV 70–100% EtOAc).

## Author contributions

CG and WK developed the hypothesis and conceptualized the study. CG, KE, SP, MF performed the experiments. CG, MF, WK analyzed the data/results. CG and WK wrote the manuscript. WK supervised the project and allocated the funding.

## Conflicts of interest

There are no conflicts to declare.

## Supplementary Material

RA-013-D2RA08005B-s001
