# Supplementary material for: Anaerobic demethylation of guaiacyl-derived monolignols enabled by a designed artificial cobalamin methyltransferase fusion enzyme
Source: RSC Adv. 2023 Feb 15;13(9):5770–7. doi: 10.1039/d2ra08005b (PMC9930637; doi:10.1039/d2ra08005b)
Supplement: RA-013-D2RA08005B-s001 [file RA-013-D2RA08005B-s001.pdf]

Electronic Supplementary Material (ESI) for RSC Advances.
This journal is © The Royal Society of Chemistry 2023

Supporting Information

Anaerobic demethylation of guaiacyl-derived monolignols enabled by a designed artificial cobalamin methyltransferase fusion enzyme

Christopher Grimm,[a] *Simona Pompei,*[a] *Kristina Egger,*[a] *Michael Fuchs,*[a] *Wolfgang Kroutil**[a,b,c]

[a] Institute of Chemistry, University of Graz – Field of Excellence BioHealth, NAWI Graz, BioTechMed Graz, Heinrichstraße 28, 8010 Graz, Austria.
[b] BioTechMed Graz, 8010 Graz, Austria
[c] Field of Excellence BioHealth, University of Graz, 8010 Graz, Austria.

*Corresponding Authors: wolfgang.kroutil@uni-graz.at

Table of contents

1. Additional data 3
   1.1 Initial experiments with preloaded methylcobalamin 3
   1.2 Operational window of reaction parameters for the fusion enzyme MT-L5-CP 4
   1.3 Substrate scope for *O*-demethylation and *O*-methylation (detailed) 8
   1.4 Activity of MT-L5-CP compared with the separate *dhaf* system 8
   1.5 One-pot reaction using Schlenk technique (25 mL scale) 10
2. Materials 11
   2.1 Plasmids used in this study 12
   2.2 DNA and amino acid sequences of used genes 13
3. Methods 18
   3.1 DNA sequence alignment of cmuA with *dhaf*-MT and *dhaf*-CP 18
   3.2 Protein production of fusion enzymes and *dhaf*-MT and *dhaf*-CP 20
   3.3 Protein purification of MT-L5-CP 20
   3.4 Determination of the pure MT-L5-CP content in the CFE 21
   3.5 Preparations of biocatalysts 21
      3.5.1 *Holo*-CP and *holo*-fusion enzyme loading 21
4. Analytics 21
   4.1 TLC and Flash Column Chromatography 21
   4.2 HPLC-UV, HPLC-MS and preparative HPLC-UV 22
      4.2.1 Method for HPLC-UV and HPLC-MS 22
      4.2.2 HPLC chromatograms 23
   4.3 NMR spectroscopy 24
      4.3.1 NMR – biotransformation of **2f** and **1c** (25 mL) 24
      4.3.2 NMRs – biotransformation of **1d** and **2a** (25 mL) 29
5. References 36

1. Additional data

1.1 Initial experiments with preloaded methylcobalamin

Table S1: Evaluation of the fusion enzymes.

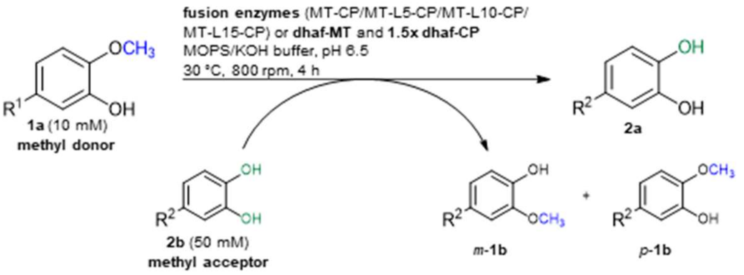

Entry	Enzyme	time [h]	2a [mM][a]	1b [mM][a]	Regioselectivity of methylation [%]
**1**	MT-CP	4	n.d.	n.d.	n.a.
		24	n.d.	n.d.	n.a.
**2**	MT-L5-CP	4	8.2	9.9	75/25 (*m*-**1b**/*p*-**1b**)
		24	8.0	9.5	72/28 (*m*-**1b**/*p*-**1b**)
**3**	MT-L10-CP	4	7.8	9.4	79/21 (*m*-**1b**/*p*-**1b**)
		24	7.9	9.6	54/46 (*m*-**1b**/*p*-**1b**)
**4**	MT-L15-CP	4	4.7	5.7	88/12 (*m*-**1b**/*p*-**1b**)
		24	7.1	8.7	82/18 (*m*-**1b**/*p*-**1b**)
**5**	MT plus 1.5xCP	4	8.5	9.9	61/39 (*m*-**1b**/*p*-**1b**)
		24	8.0	9.5	49/51 (*m*-**1b**/*p*-**1b**)

[a] The discrepancy between methylation and demethylation can be explained by the prior methylcobalamin loading step, which enables the initial methylation of the methyl acceptor. Reaction conditions: methyl donor **1a** (10 mM, 1.2 mg/mL), methyl acceptor **2b** (50 mM, 9 mg/mL), MT-X-CP (Entries 1-4, 208 mg/mL CFE, ≡ 37 mg/mL, 0.6 mM pure MT-L5-CP) or MT+1.5xCP (Entry 5, each 104 mg/mL CFE for *dhaf*-MT, ≡ 34 mg/mL,0.9 mM pure *dhaf*-MT[1] and *dhaf*-CP, ≡ 22 mg/mL, 1 mM pure *dhaf*-CP[2]) in MOPS buffer (50 mM, pH 6.5, 150 mM KCl) at 30 °C, 800 rpm in Eppendorf Thermomixer® (1.5 mL), in a glovebox (N_2) for 4 h and 24 h. total volume: 120 µL. All reactions were quenched by the addition of MeCN (60 vol. % final conc.) after 4 h and the conversions were analyzed via calibration curves of the corresponding reference compounds on HPLC-UV. n.d. not detected. n.a. not applicable.

1.2 Operational window of reaction parameters for the fusion enzyme MT-L5-CP

Varied methylcobalamin concentrations avoiding an extra loading step

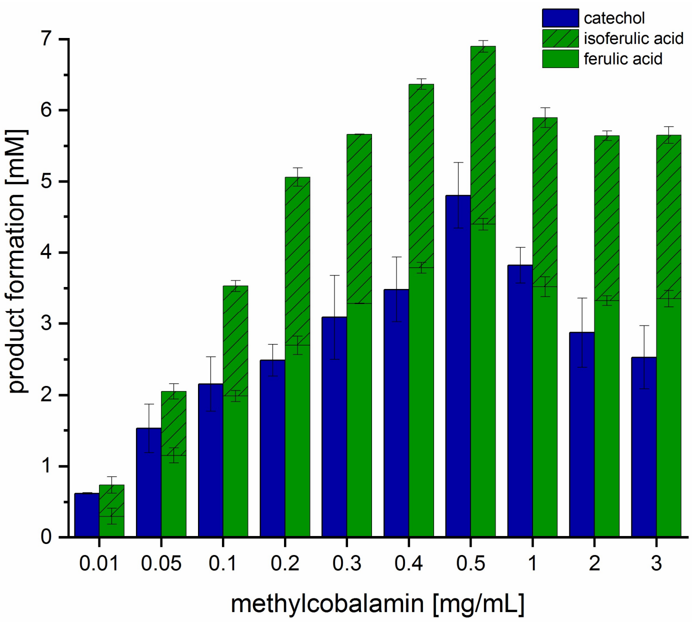

Figure S1. Varied methylcobalamin concentrations (0.01-3 mg/mL, 7.4 µM-2.2 mM). Reaction conditions: methyl donor **1a** (10 mM, 1.2 mg/mL), methyl acceptor **2b** (50 mM, 9 mg/mL), MT-L5-CP (208 mg/mL CFE, ≡ 37 mg/mL, 0.6 mM pure MT-L5-CP) in MOPS buffer (50 mM, pH 6.5, 150 mM KCl) with different MeCob concentrations (0.01-3 mg/mL, 7.4 µM-2.2 mM) at 30 °C, 800 rpm in Eppendorf Thermomixer® (1.5 mL) for 4 h. total volume: 120 µL. All reactions were quenched by the addition of MeCN (60 vol. % final conc.) after 4 h and the conversions were analyzed via calibration curves of the corresponding reference compounds on HPLC-UV.

Ratio methyl donor and acceptor

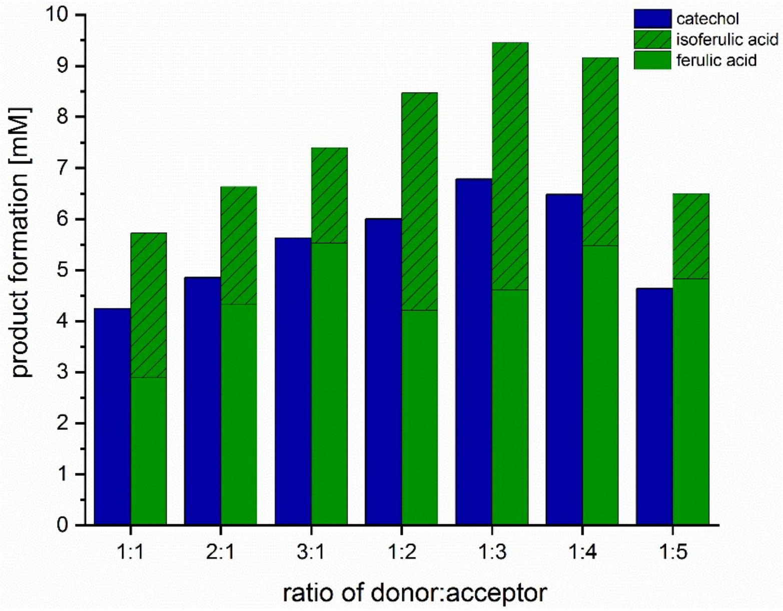

Figure S2. Study on varied ratios between methyl donor and acceptor. Reaction conditions: methyl donor **1a** (10-30 mM, 1.2-3.6 mg/mL), methyl acceptor **2b** (10-50 mM, 1.8-9 mg/mL), MT-L5-CP (208 mg/mL CFE, ≡ 0.6 mM pure MT-L5-CP) in MOPS buffer (50 mM, pH 6.5, 150 mM KCl) with MeCob (0.5 mg/mL, 0.37 mM) at 30 °C, 800 rpm in Eppendorf Thermomixer® (1.5 mL) for 4 h. total volume: 120 µL. All reactions were quenched by the addition of MeCN (60 vol. % final conc.) after 4 h and the conversions were analyzed via calibration curves of the corresponding reference compounds on HPLC-UV.

Variation of amount of CFE employed

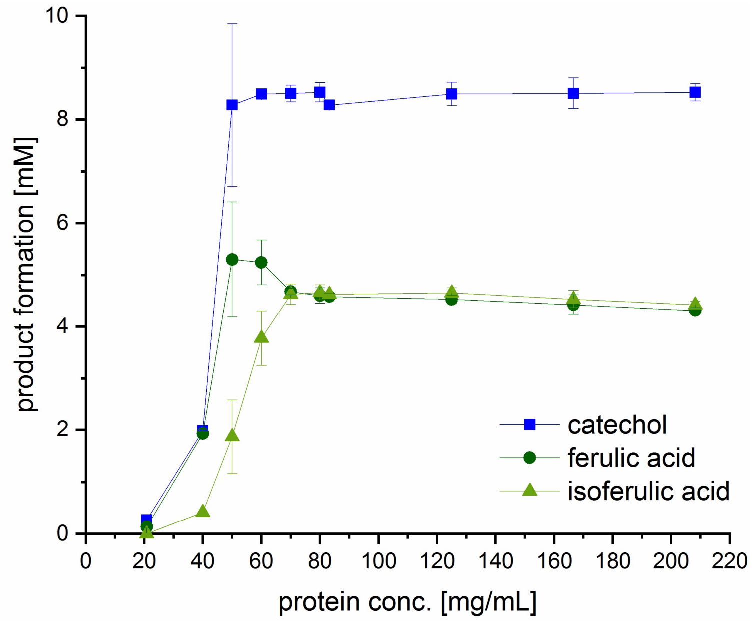

Figure S3. Varied concentration of total protein of CFE containing MT-L5-CP (20.8 mg/mL -208 mg/mL). Reaction conditions: methyl donor **1a** (10 mM, 1.2 mg/mL), methyl acceptor **2b** (30 mM, 5.4 mg/mL), MT-L5-CP (20.8- 208 mg/mL CFE, ≡ 0.06- 0.6 mM pure MT-L5-CP) in MOPS buffer (50 mM, pH 6.5, 150 mM KCl) with MeCob (0.5 mg/mL, 0.37 mM) at 30 °C, 800 rpm in Eppendorf Thermomixer® (1.5 mL) for 4 h. total volume: 120 µL. All reactions were quenched by the addition of MeCN (60 vol. % final conc.) after 4 h and the conversions were analyzed via calibration curves of the corresponding reference compounds on HPLC-UV.

Table S2. Varied concentration of CFE containing MT-L5-CP (20.8 mg/mL -208 mg/mL). Reaction conditions: methyl donor **1a** (10 mM, 1.2 mg/mL), methyl acceptor **2b** (30 mM, 5.4 mg/mL), MT-L5-CP (20.8- 208 mg/mL CFE, ≡ 3.7- 37 mg/mL, 0.06- 0.6 mM pure MT-L5-CP) in MOPS buffer (50 mM, pH 6.5, 150 mM KCl) with MeCob (0.5 mg/mL, 0.37 mM) at 30 °C, 800 rpm in Eppendorf Thermomixer® (1.5 mL) for 4 h. total volume: 120 µL. All reactions were quenched by the addition of MeCN (60 vol. % final conc.) after 4 h and the conversions were analyzed via calibration curves of the corresponding reference compounds on HPLC-UV. Data corresponds to Figure S3.

		Demethylation	Methylation	
Equivalent of pure MT-L5-CP [mM]	MT-L5-CP $[mg/mL]_{CFE}$	**2a** [mM]	*m*-**1b** [mM]	*p*-**1b** [mM]
0.06	21	0.3	0.1	0
0.12	40	2.0	1.9	0.4
0.14	50	8.3	5.3	1.9
0.17	60	8.5	5.2	3.8
0.20	70	8.5	4.7	4.6
0.23	80	8.3	4.6	4.6
0.24	83	8.5	4.6	4.6
0.36	125	8.5	4.5	4.6
0.48	167	8.5	4.4	4.5
0.6	208	8.5	4.3	4.4

Methyl transfer at varied amount of purified MT-L5-CP

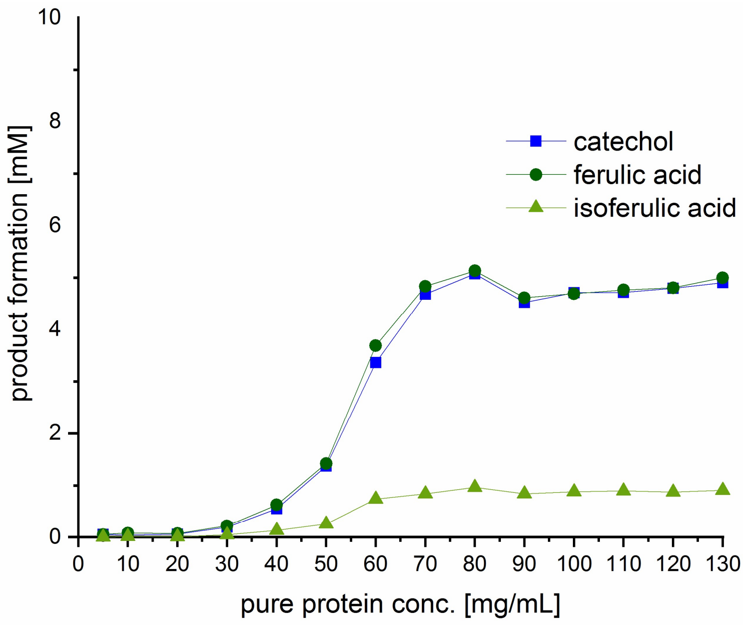

Figure S4. Product formation at varied protein concentrations of His-tag purified MT-L5-CP (5 mg/mL -130 mg/mL). Reaction conditions: methyl donor **1a** (10 mM, 1.2 mg/mL), methyl acceptor **2b** (30 mM, 5.4 mg/mL), pure MT-L5-CP (5 mg/mL -130 mg/mL, 0.08 mM – 2.2 mM) in MOPS buffer (50 mM, pH 6.5, 150 mM KCl) with MeCob (0.5 mg/mL, 0.37 mM) at 30 °C, 800 rpm in Eppendorf Thermomixer® (1.5 mL) for 4 h. total volume: 120 µL. All reactions were quenched by the addition of MeCN (60 vol. % final conc.) after 4 h and the conversions were analyzed via calibration curves of the corresponding reference compounds on HPLC-UV.

Table S3. Product formation at varied protein concentrations of His-tag purified MT-L5-CP (5 mg/mL -130 mg/mL). Reaction conditions: methyl donor **1a** (10 mM, 1.2 mg/mL), methyl acceptor **2b** (30 mM, 5.4 mg/mL), pure MT-L5-CP (5 mg/mL -130 mg/mL, 0.08 mM – 2.2 mM) in MOPS buffer (50 mM, pH 6.5, 150 mM KCl) with MeCob (0.5 mg/mL, 0.37 mM) at 30 °C, 800 rpm in Eppendorf Thermomixer® (1.5 mL) for 4 h. total volume: 120 µL. All reactions were quenched by the addition of MeCN (60 vol. % final conc.) after 4 h and the conversions were analysed via calibration curves of the corresponding reference compounds on HPLC-UV. Data corresponds to Figure S4.

		Demethylation	Methylation	
MT-L5-CP [mM]	MT-L5-CP $[mg/mL]_{pure}$	**2a** [mM]	*m*-**1b** [mM]	*p*-**1b** [mM]
0.085	5	0.05	0.05	0
0.17	10	0.04	0.08	0.01
0.33	20	0.06	0.07	0.01
0.51	30	0.2	0.2	0.1
0.68	40	0.5	0.6	0.3
0.85	50	1.4	1.4	0.7
1.02	60	3.4	3.7	0.8
1.18	70	4.7	4.8	1.0
1.35	80	5.1	5.1	0.8
1.52	90	4.5	4.6	0.8
1.69	100	4.7	4.7	0.9
1.86	110	4.7	4.8	0.9
2.03	120	4.8	4.8	0.9
2.2	130	4.9	5.0	0.9

Time course of methyl transfer

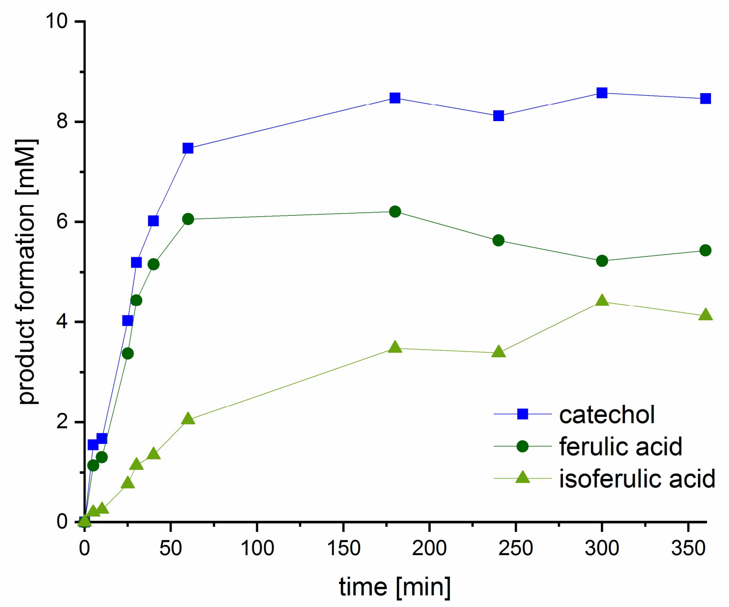

Figure S5. Time study of the biotransformation of the fusion enzyme MT-L5-CP. Reaction conditions: methyl donor **1a** (10 mM, 1.2 mg/mL), methyl acceptor **2b** (30 mM, 5.4 mg/mL), MT-L5-CP (60 mg/mL CFE, ≡ 11 mg/mL, 0.18 mM pure MT-L5-CP) in MOPS buffer (50 mM, pH 6.5, 150 mM KCl) with MeCob (0.5 mg/mL, 0.37 mM) at 30 °C, 800 rpm in Eppendorf Thermomixer® (1.5 mL) for 5 min to 360 min. total volume: 120 µL. All reactions were quenched by the addition of MeCN (60 vol. % final conc.) and the conversions were analyzed via calibration curves of the corresponding reference compounds on HPLC-UV.

1.3 Substrate scope for *O*-demethylation and *O*-methylation (detailed)

Table S4. Substrate scope of MT-L5-CP for the *O*-demethylation of guaiacol derivatives and the *O*-methylation of catechol derivatives.

Entry	Substrate	Co-substrate	Demethylated product **2** [%]	TON **2**	Methylated products **1** [%]	Regioselectivity [%]
1	**1a**	**2b**	86± 6	48	82± 5	65/35 (*m*-**1b**/*p*-**1b**)
2	**1a**	**2e**	77± 2	43	73± 1	68/32 (*m*-**1e**/*p*-**1e**)
3	**1a**	**2f**	85± 1	47	82± 1	46/54 (*m*-**1f**/*p*-**1f**)
4	*m*-**1b**	**2f**	67± 3	37	60± 1	48/52 (*m*-**1f**/*p*-**1f**)
5	*m*-**1b**	**2a**	68± 1	38	57± 3	100 **1a**
6	**1c**	**2e**	86± 5	48	76± 1	70/30 (*m*-**1e**/*p*-**1e**)
7	**1c**	**2f**	96± 2	53	85± 2	47/53 (*m*-**1f**/*p*-**1f**)
8	**1c**	**2a**	94± 3	52	81± 3	100 **1a**
9	**1d**	**2b**	68± 1	38	56± 1	84/26 (*m*-**1b**/*p*-**1b**)
10	**1d**	**2a**	83± 2	46	70± 4	100 **1a**

Reaction conditions: substrate **1a**, *m*-**1b**, **1c-d** (10 mM), co-substrate **2a-b**, **2e-f** (30 mM), methyl donors **1a-e** (10 mM), MT-L5-CP (60 mg/mL CFE corresponds to 11 mg/mL, 0.18 mM pure MT-L5-CP) in MOPS buffer (50 mM, pH 6.5, 150 mM KCl) with MeCob (0.5 mg/mL, 0.37 mM) at 30 °C, 800 rpm in Eppendorf Thermomixer (1.5 mL) for 3 h under inert atmosphere (glovebox). total volume: 120 µL. All reactions were quenched by the addition of MeCN (60 vol. % final conc.) and the conversions were analyzed via calibration curves of the corresponding reference compounds on HPLC-UV.

The turnover number (TON) for all demethylated products was calculated using the following equation:

$$TON = \frac{total\ product\ formation\ (mM)}{amount\ of\ catalyst\ (mM)} \qquad (1)$$

1.4 Activity of MT-L5-CP compared with the separate *dhaf* system

To determine the activity of the MT-L5-CP and compare this with the separate *dhaf* system, all CFE samples were analyzed by SDS-PAGE (Figure S6) and adjusted on an equal protein content. Therefore, MT-L5-CP was taken as template (lane 1, 9.2 pmol) and the amount of *dhaf*-MT (lane 2, 12.6 pmol) as well as *dhaf*-CP (lane 3, 19.3 pmol) were normalized.

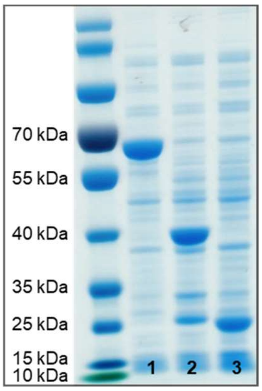

Figure S6. SDS-PAGE of protein preparations of MT-L5-CP (lane 1), *dhaf*-MT (lane 2) and *dhaf*-CP (lane 3). All proteins were applied with 10 µg protein per lane.

Regarding the biocatalytic reaction, best substrates 2-methoxy-5-methylphenol **1c** (10 mM donor) and 3,4-dihydroxybenzyl alcohol **2f** (30 mM acceptor) and optimized conditions were used. The protein concentration of MT-L5-CP was lowered (from 60 mg/mL to 10 mg/mL) due to high product formation for both methylation (85% *m*-**1f** and *p*-**1f**) and demethylation (96% **2c**). The initial rate of the methylated products vanillyl alcohol *m*-**1f** and isovanillyl alcohol *p*-**1f** as well as the demethylated product 4-methylcatechol **2c** were analyzed within 20 min on HPLC-UV for both MT-L5-CP (Figure S7) and the *dhaf* system (Figure S8).

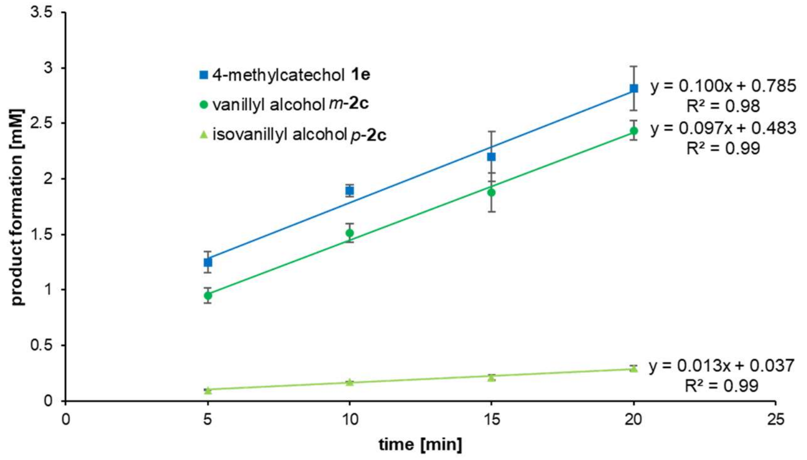

Figure S7. Initial rates of the methylation and demethylation with MT-L5-CP. The demethylated product 4-methylcatechol **2c** (square) and methylated products vanillyl alcohol *m*-**1f** (dots) and isovanillyl alcohol *p*-**1f** (triangles) were analyzed within 20 min and detected on HPLC-UV. Reaction conditions: methyl donor **1c** (10 mM), methyl acceptor **2f** (30 mM), MT-L5-CP (10 mg/mL CFE corresponds to 1.8 mg/mL, 0.03 mM pure MT-L5-CP) in MOPS buffer (50 mM, pH 6.5, 150 mM KCl) with MeCob (0.5 mg/mL, 0.37 mM) at 30 °C, 800 rpm in Eppendorf Thermomixer (1.5 mL) for 3 h. total volume: 120 µL. All reactions were quenched by the addition of MeCN (60 vol. % final conc.) and the conversions were analyzed via calibration curves of the corresponding reference compounds on HPLC-UV.

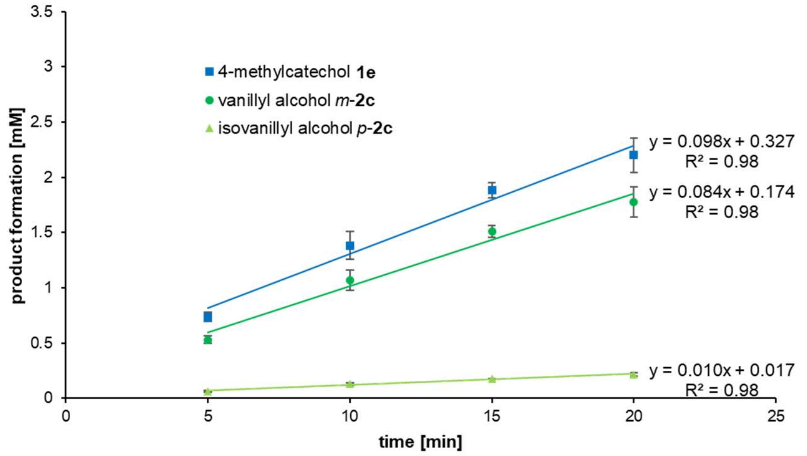

Figure S8. Initial rates of the methylation and demethylation with the separate *dhaf* system. The demethylated product 4-methylcatechol **2c** (square) and methylated products vanillyl alcohol *m*-**1f** (dots) and isovanillyl alcohol *p*-**1f** (triangles) were analyzed within 20 min and detected on HPLC-UV. Reaction conditions: methyl donor **1c** (10 mM), methyl acceptor **2f** (30 mM), MT-L5-CP (10 mg/mL CFE corresponds to 1.8 mg/mL, 0.03 mM pure MT-L5-CP) in MOPS buffer (50 mM, pH 6.5, 150 mM KCl) with MeCob (0.5 mg/mL, 0.37 mM) at 30 °C, 800 rpm in Eppendorf Thermomixer (1.5 mL) for 3 h. total volume: 120 µL. All reactions were quenched by the addition of MeCN (60 vol. % final conc.) and the conversions were analyzed via calibration curves of the corresponding reference compounds on HPLC-UV.

1.5 One-pot reaction using Schlenk technique (25 mL scale)

The fusion enzyme MT-L5-CP was applied on a semi-preparative scale using Schlenk technique to catalyze the *O*-demethylation of 2-methoxy-5-methylphenol **1c** or coniferyl alcohol **1d** with the concomitant *O*-methylation of the co-substrates 3,4-dihydroxybenzyl alcohol **2f** or catechol **2a**. Here, detailed information for both methyl transfer reactions are depicted in Figure S9:

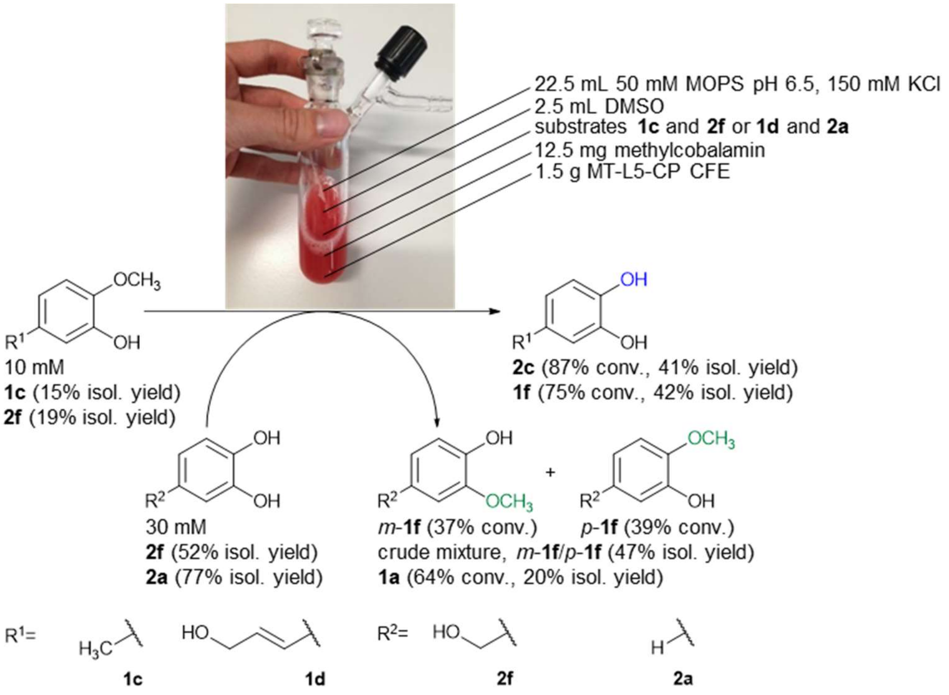

Figure S9. Semi-preparative methyl transfer of 1c or 1d with 2f or 2a catalyzed by MT-L5-CP. Reaction conditions: substrate **1c** or **1d** (10 mM), co-substrate **2f** or **2a** (30 mM), MT-L5-CP (60 mg/mL CFE corresponds to 11 mg/mL, 0.18 mM pure MT-L5-CP) in MOPS buffer (50 mM, pH 6.5, 150 mM KCl) with MeCob (0.5 mg/mL, 0.37 mM) at 30 °C and 160 rpm BioInfor shaker for 3 h under inert atmosphere (Ar, Schlenk line). total volume: 25 mL. All reactions were quenched by the addition of MeCN (60 vol. % final conc.) and the conversions were analyzed via calibration curves of the corresponding reference compounds on HPLC-UV.

2. Materials

All substrates, solvents, antibiotics and supplementary materials (e.g. LB-media) available in the laboratory were obtained from various suppliers (Acros Organics, Alfa Aesar, Sigma-Aldrich, TCI-Chemicals or VWR International/Merck and Roth) in highest available purities and used as received unless stated otherwise.

The following compounds were obtained by the mentioned suppliers and used as corresponding references for HPLC-UV qualification: caffeic acid **2b**, ferulic acid *m*-**1b**, isoferulic acid *m*-**1b**, 3,4-dihydroxybenzaldehyde **2e**, vanillin *m*-**1e**, isovanillin *p*-**1e**, 3,4-dihydroxybenzyl alcohol **2f**, vanillyl alcohol *m*-**1f**, isovanillyl alcohol *p*-**1f**, catechol **2a**, guaiacol **1a**, 2-methoxy-5-methylphenol **1c**, 4-methylcatechol **2c**, coniferyl alcohol **1d** and caffeoyl alcohol **2d** as well as sinapyl alcohol **1g** and *p*-coumaryl alcohol **1h**.

Structure of sinapyl alcohol (**1g**)

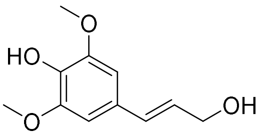

sinapyl alcohol **1g**

All synthetic genes, encoding for the fusion enzymes, were ordered already cloned in pET28a(+) from the supplier (General Biosystems). Regarding protein production, cell cultures were shaken in a HT Infors Unitron AJ260 incubator and harvested at 4 °C by centrifugation using either a Hitachi Centrifuge CR22N (4,000-14,000 rpm, 7,520-33,600 rcf) or Hettich Centrifuge Rotina 420R (4,000 rpm, 4,000 rcf). To disrupt the cells the ultrasonication with a Branson Digital Sonifier was used. The optical cell density OD600 (λ= 600 nm) and the protein concentration was measured at an Eppendorf Bio Photometer Plus. All biocatalytic reaction samples (120 or 500 µL) were shaken in a 1.5 mL Eppendorf Thermomixer performed under oxygen-free atmosphere using a MBraun LABstar glovebox (99.8% N_2, 5 bar) equipped with O2-sensor (MB-OX-EC) for aqueous solutions. Work-up of biotransformation samples were performed under aerobic atmosphere using an Eppendorf Microcentrifuge (rt, 14,000 rpm, 15,800 rcf).

2.1 Plasmids used in this study

Table S5. Empty plasmids and plasmids with genes encoding for enzymes from *Desulfitobacterium hafniense*

Plasmids[a]	Origin (GeneBank ID)	Description/Comments
pASK-IBA3plus	IBA-Lifescience	P_{Tet}, Amp^r, $ColE1_{ori}$, *C*-terminal StrepTag
pEG457	literature[1,3–5]	*dhaf*-MT, methyltransferase from *Desulfitobacterium hafniense*, codon optimised gene cloned into pASK-IBA3plus, *EcoRI* & *HindIII*
pEG459	literature[1,3–5]	*dhaf*-CP, corrinoid-binding protein from *Desulfitobacterium hafniense*, codon optimised gene cloned into pASK-IBA3plus, *EcoRI* & *HindIII*
pET21a(+)	Novagen	P_{T7lac}, Amp^r, $pBR322_{ori}$, *N*- and/or *C*-terminal HisTag
pEG465	this study	cmuA, natural fusion enzyme (MT and CP) from *Hyphomicrobium chloromethanicum*, codon-optimised gene cloned into pET21a(+), *NdeI* & *HindIII*
pET28a(+)	Novagen	P_{T7lac}, Kan^r, $pBR322_{ori}$, thrombin-cleavage site, *N*- terminal HisTag and/or *C*-terminal HisTag
pEG599	this study	MT-CP, fusion enzyme of *dhaf*-MT and *dhaf*-CP from *D. hafniense*, wild-type gene was ordered from General Biosystems cloned in pET28a(+), *NdeI* & *XhoI*
pEG600	this study	MT-L5-CP, fusion enzyme with GGGGS linker (*Eco*RI, *Hind*III) between *dhaf*-MT and *dhaf*-CP from *D. hafniense*, gene was ordered from General Biosystems cloned in pET28a(+), *NdeI* & *XhoI*
pEG601	this study	MT-L10-CP, fusion enzyme with GGGGSGGGGS linker (*Eco*RI, *Hind*III) between *dhaf*-MT and *dhaf*-CP from *D. hafniense*, gene was ordered from General Biosystems cloned in pET28a(+), *NdeI* & *XhoI*
pEG602	this study	MT-L15-CP, fusion enzyme with GGGGSGGGGSGGGGS linker (*Eco*RI, *Hind*III) between *dhaf*-MT and *dhaf*-CP from *D. hafniense*, gene was ordered from General Biosystems cloned in pET28a(+), *NdeI* & *XhoI*

[a] pEG number is an internal numbering of plasmids (stands for **p**lasmid of the **E**lk **G**roup)

2.2 DNA and amino acid sequences of used genes

Dhaf4610 (dhaf-MT) in pASK-IBA3plus:

DNA sequence

gaattc**ATG**CTGACCATTAAACAGAATCTGCTGGAAACCATTCGTGGTGGTAATCCG-
GATCGTTTTGTTAATCAGTATGAGTTCATGGATATCATCCTGGAAAATCCGGTTGATTTTATGGCACCGCCTGG
TGGTAGCATTAAAACCGGTTGGGGTATTACCTTTCGTTGGCCTGAAGGTCAGATTGGTCAGTTTCCGGTTCATG
ATGAAGAACACAAAGTGCTGAAAGACATTACCAAATGGCGTGAACAGGTTAAAGCACCGGAAATTCCGGATA
CCGATGAAGCATGGGCAGCAGCAGTTGAACATGCAAATAGCATTGATCGCAACGAGAAATATGTTACCGCATT
TGTTGCACCGGGTGTTTTTGAAATGTGTCATCATCTGATGAGCATGGAAGATGCACTGATGGCACTGTATGAA
GAACCTGAACTGATGCATGAACTGATCGATTATCTGACCGAATATGAACTGAAACTGGCCGAAATTATCTGTC
GTCGTCTGAAACCGGATGCACTGTTTCATCACGATGATTGGGGTAGCCAGAAAAGCAGCTTTATTAGTCCGGC
AATGTTCGAAGAATTTTATCTGCCTGCCTACAAAAAAATCTATAGCTTCTATAAACAGAACGGCGTTGAACTG
ATTGTGCATCATAGCGATTGTTATGCAGCAAATCTGGTGCCGTTTATGATTGAAATGGGCATTGATATTTGGCA
GGGTGTTATGACCACCAATAATACACCGGAACTGATTAAACAGTACGGTGGCAAAATTAGCTTTATGGGCGAT
ATTGATAGCGGTGTTGTTGATTTTCCGACCTGGACCCGTGAAATTGCAGCACGTGAAGCAGAACGTGCATGTA
CCAATTGTGGTAAACATTATTTCATTCCGTGTCTGACCCAGGGTCTGGGTTTTAGCAGCTTTCCGGGTGTGTAT
GATTGTGTTAGCGAAGAAATTGATAAACTGAGCAAAAAAATGTTCGTCGAC**TAA**ctcgag

gaattc= *EcoRI*, ctcgag= *XhoI*

Amino acid sequence

MLTIKQNLLETIRGGNPDRFVNQYEFMDIILENPVDFMAPPGGSIKTGWGITFRWPEGQIGQFPVHDEEHKVLKDITK
WREQVKAPEIPDTDEAWAAAVEHANSIDRNEKYVTAFVAPGVFEMCHHLMSMEDALMALYEEPELMHELIDYLT
EYELKLAEIICRRLKPDALFHHDDWGSQKSSFISPAMFEEFYLPAYKKIYSFYKQNGVELIVHHSDCYAANLVPFMIE
MGIDIWQGVMTTNNTPELIKQYGGKISFMGDIDSGVVDFPTWTREIAAREAERACTNCGKHYFIPCLTQGLGFSSFP
GVYDCVSEEIDKLSKKMFVD*

Dhaf4611 (dhaf-CP) in pASK-IBA3plus:

DNA sequence

gaattc**ATG**AGCAAAATCGCCGAAGTTAAAGCAATGGTTGAAGCAGGTAAA-
GCAAAACTGGTTCCGGGTCTGGTTCAAGAGGCACTGGATGCAGGTAATGCAGCCGGTGATATTCTGGCAGGTA
TGATTGATAGCATGGGTGTTGTTGGTGATAAATTCAGTGCCGGTGAACTGTTTGTTCCGGAAATGCTGATGGCA
GCAAAAGCAATGAGCAAAGGTGTTGATGTTCTGAAACCGCATCTGACCGGTGAAAGCGCAACCAGCCTGGGC
ACCTGTGTTATTGGCACCGTTGCCGGTGATCTGCATGATATTGGTAAAAATCTGGTTGCCATGATGCTGGAAAG
CGTTGGTTTTAATGTTGTTGATCTGGGTGTGGATGTTAGCGCAGAAAAATTTGTTGATGCCGTGCGCGAAAATG
ACAACGTTAAAATTGTTGCATGTAGCGGTCTGCTGACCACCACCATGCCTGCAATGAAAGAAACCGTTCAGAG
CCTGAAAAATTCAGGTCTGACCGGCTTTAAAGTTATTGTTGGTGGTGCACCGGTTAGCCAGGCAATGGCAGAT
GAAATTGGTGCAGATGGTTTTGCACCGGATGCCGGTGGTGCAGCAGTTAAAGCCAAAGAACTGGCACATGCA
GTCGAC**TAA**ctcgag

gaattc= *EcoRI*, ctcgag= *XhoI*

Amino acid sequence

MSKIAEVKAMVEAGKAKLVPGLVQEALDAGNAAGDILAGMIDSMGVVGDKFSAGELFVPEMLMAAKAMSKGVD
VLKPHLTGESATSLGTCVIGTVAGDLHDIGKNLVAMMLESVGFNVVDLGVDVSAEKFVDAVRENDNVKIVACSGL
LTTTMPAMKETVQSLKNSGLTGFKVIVGGAPVSQAMADEIGADGFAPDAGGAAVKAKELAHAVD*

cmuA (MT and CP from Hyphomicrobium chloromethanicum) in pET21a(+):

DNA sequence

cat**ATG**ACCCAGGTTCCGAAAATGACCAGCCGTGAACGTCTGTTTGCAGCAGTTAC-
CATGCAGACCCTGCCGGATCAGGTTCCGTGTGTTCCGCTGCTGATGACCCGTGGTATTCGTGAAGGTGGTATTA
CCGTTGATCAGGCACTGCGTGATGGTGAAGCAAGCGCACATGCAAAAATCAAAGCACTGAAAAAATTCGGTG
GCGACGTTATTATTCCGGGTACAGACCTGTTTACACCGGTTGAATGTGTTGAAGGTTGTGAACTGGATTATCTG
CCGTATGCACAGCCGAGCCTGGTTAAACATCCGACCCCGACCAAAGAAGCATTTTATCGCTATAAAGAGAAAT
ATCTGCGCGAAGGTTTTAAACCGAGCGAACGTGTTCTGCAGATTCAGCGTGCACGTACCATGATTGCACGTCG
TAAAGATACCCATGCAATGCCGACACCGGTGGGTGGTCCGATTACCCGTGCACAGCTGATGACAGGTAGCAGC
GAATTTCTGAGCTATATTAGTGATGATCCGGATTATGCCAAAGAAGTTACCGAACTGGCACTGGATATTGTGA
AAAATGTTTGCCGCATGATGTTTGAAGCCGGTATTGATGTTTGCAATATCCTGGATCCGTTTAACAGCAGCGAT
ATTCTGCCTCCGGATACCTATCGTGAATTTGGCCTGCCGTATCAGAAACGCCTGTTTGCCTATATCAAAGAAAT
TGGTGGTATCGGCTTTACCCATACCTGTACCTTTACCCAGCCGATTTGGCGTGATATTGCCAATAATGGTTGCT
TTAACTTCAACGGCGATATGTATCCTGGTATGGATCATGCAAAACGTGCCATTGGTGGTCAGATTAGCCTGATG
GGCACCCTGAGCCCGTTTAGCACCTTTATGCATGGTTGGACCACCGATGTTGCGAACAAAGTTAAAAAACTGG
CAGCCGAAGTTGGTTATAATGGTGGTCTGATTGTTATGCCTGGTTGCGATATTGATTGGACCATTCCGGATGAA
AATCTGAAAGCAATGATTGAAACCTGCGCCAGCATCAAATATCCGATGGATGTTGCAGCACTGGGTGATCTGA
GCAATGTTTATCTGGCAGGTCATCCGAAACATCCGGGTAAACGTGCACCGAGCACCGCAGGCGATACCGATGT
GGCAGAAGCAAAAACCCATCATAAAGAACTGACACCGCAGCAAGAAGTGAACGAAAAACTGGTTGAAGCCAT
CATGGAATATGATGGCGATAAAGCAATTGAATGGGTGAAAAAAGGTCTGGAACGTGGTATGACCGCACAGGA
TATCGTTTTTGATGGTCTGAGCCTGGGTATGAAAGTTGTTGGTGATATGTATGAACGCAACGAACGTTTTGTTA
CCGATATGCTGAAAGCAGCCAAAACAATGGATAAAGCCATGCCGATTCTGACCCCTCTGCTGGAACAGGCAG
GCGGTGATGGCGGTCCGACCGGCACCGTTGTTGTTGGTCTGGTTCGTGGTAATACCCAGGACATTGGTAAAAA
TCTGGTTTGCCTGATGCTGAAAGCGAATGGCTTTAAAGTTATTGACCTGGGCAAAAACGTTAAACCGGAACAG
TTTATTGAAAGCGCAGAAAAAGAAAATGCCGTGGCAATTGGTATGAGCGTTATGACCAATAGCAGCACCGTTT
ATGTGGAAAAAGTGAAAGAACTGCTGGACAAAGCAGGTAAAGGTGATAAATACCTGCTGATGTGTGGTGGTG
CAGCAGCAAATAAAGGTGTTGCAGATAAAATGGGTGTGAAATATGGTCTGGATGCAAATGCAGCCGTTAGCCT
GGTGAAAGATCATCTGCAGGCAGCAGCACTCGAG**TAA**gctt

cat**ATG**= *NdeI*, **AA**gctt= *HindIII*

Amino acid sequence

MTQVPKMTSRERLFAAVTMQTLPDQVPCVPLLMTRGIREGGITVDQALRDGEASAHAKIKALKKFGGDVIIPGTDL
FTPVECVEGCELDYLPYAQPSLVKHPTPTKEAFYRYKEKYLREGFKPSERVLQIQRARTMIARRKDTHAMPTPVGG
PITRAQLMTGSSEFLSYISDDPDYAKEVTELALDIVKNVCRMMFEAGIDVCNILDPFNSSDILPPDTYREFGLPYQKR
LFAYIKEIGGIGFTHTCTFTQPIWRDIANNGCFNFNGDMYPGMDHAKRAIGGQISLMGTLSPFSTFMHGWTTDVAN
KVKKLAAEVGYNGGLIVMPGCDIDWTIPDENLKAMIETCASIKYPMDVAALGDLSNVYLAGHPKHPGKRAPSTAG
DTDVAEAKTHHKELTPQQEVNEKLVEAIMEYDGDKAIEWVKKGLERGMTAQDIVFDGLSLGMKVVGDMYERNE
RFVTDMLKAAKTMDKAMPILTPLLEQAGGDGGPTGTVVVGLVRGNTQDIGKNLVCLMLKANGFKVIDLGKNVKP
EQFIESAEKENAVAIGMSVMTNSSTVYVEKVKELLDKAGKGDKYLLMCGGAAANKGVADKMGVKYGLDANAA
VSLVKDHLQAAALE*

MT-CP (dhaf-MT-dhaf-CP)

DNA sequence

catatg**ATG**CTGACCATTAAACAGAATCTGCTGGAAACCATTCGTGGTGGTAATCCG-
GATCGTTTTGTTAATCAGTATGAGTTCATGGATATCATCCTGGAAAATCCGGTTGATTTTATGGCACCGCCTGG
TGGTAGCATTAAAACCGGTTGGGGTATTACCTTTCGTTGGCCTGAAGGTCAGATTGGTCAGTTTCCGGTTCATG
ATGAAGAACACAAAGTGCTGAAAGACATTACCAAATGGCGTGAACAGGTTAAAGCACCGGAAATTCCGGATA
CCGATGAAGCATGGGCAGCAGCAGTTGAACATGCAAATAGCATTGATCGCAACGAGAAATATGTTACCGCATT
TGTTGCACCGGGTGTTTTTGAAATGTGTCATCATCTGATGAGCATGGAAGATGCACTGATGGCACTGTATGAA

GAACCTGAACTGATGCATGAACTGATCGATTATCTGACCGAATATGAACTGAAACTGGCCGAAATTATCTGTC
GTCGTCTGAAACCGGATGCACTGTTTCATCACGATGATTGGGGTAGCCAGAAAAGCAGCTTTATTAGTCCGGC
AATGTTCGAAGAATTTTATCTGCCTGCCTACAAAAAAATCTATAGCTTCTATAAACAGAACGGCGTTGAACTG
ATTGTGCATCATAGCGATTGTTATGCAGCAAATCTGGTGCCGTTTATGATTGAAATGGGCATTGATATTTGGCA
GGGTGTTATGACCACCAATAATACACCGGAACTGATTAAACAGTACGGTGGCAAAATTAGCTTTATGGGCGAT
ATTGATAGCGGTGTTGTTGATTTTCCGACCTGGACCCGTGAAATTGCAGCACGTGAAGCAGAACGTGCATGTA
CCAATTGTGGTAAACATTATTTCATTCCGTGTCTGACCCAGGGTCTGGGTTTTAGCAGCTTTCCGGGTGTGTAT
GATTGTGTTAGCGAAGAAATTGATAAACTGAGCAAAAAAATGTTCGTCGACATGAGCAAAATCGCCGAAGTT
AAAGCAATGGTTGAAGCAGGTAAAGCAAAACTGGTTCCGGGTCTGGTTCAAGAGGCACTGGATGCAGGTAAT
GCAGCCGGTGATATTCTGGCAGGTATGATTGATAGCATGGGTGTTGTTGGTGATAAATTCAGTGCCGGTGAAC
TGTTTGTTCCGGAAATGCTGATGGCAGCAAAAGCAATGAGCAAAGGTGTTGATGTTCTGAAACCGCATCTGAC
CGGTGAAAGCGCAACCAGCCTGGGCACCTGTGTTATTGGCACCGTTGCCGGTGATCTGCATGATATTGGTAAA
AATCTGGTTGCCATGATGCTGGAAAGCGTTGGTTTTAATGTTGTTGATCTGGGTGTGGATGTTAGCGCAGAAAA
ATTTGTTGATGCCGTGCGCGAAAATGACAACGTTAAAATTGTTGCATGTAGCGGTCTGCTGACCACCACCATG
CCTGCAATGAAAGAAACCGTTCAGAGCCTGAAAAATTCAGGTCTGACCGGCTTTAAAGTTATTGTTGGTGGTG
CACCGGTTAGCCAGGCAATGGCAGATGAAATTGGTGCAGATGGTTTTGCACCGGATGCCGGTGGTGCAGCAGT
TAAAGCCAAAGAACTGGCACATGCAGTCGAC**TAA**ctcgag

catatg= *NdeI*, ctcgag= *XhoI*

Amino acid sequence

MLTIKQNLLETIRGGNPDRFVNQYEFMDIILENPVDFMAPPGGSIKTGWGITFRWPEGQIGQFPVHDEEHKVLKDITK
WREQVKAPEIPDTDEAWAAAVEHANSIDRNEKYVTAFVAPGVFEMCHHLMSMEDALMALYEEPELMHELIDYLT
EYELKLAEIICRRLKPDALFHHDDWGSQKSSFISPAMFEEFYLPAYKKIYSFYKQNGVELIVHHSDCYAANLVPFMIE
MGIDIWQGVMTTNNTPELIKQYGGKISFMGDIDSGVVDFPTWTREIAAREAERACTNCGKHYFIPCLTQGLGFSSFP
GVYDCVSEEIDKLSKKMFVDMSKIAEVKAMVEAGKAKLVPGLVQEALDAGNAAGDILAGMIDSMGVVGDKFSAG
ELFVPEMLMAAKAMSKGVDVLKPHLTGESATSLGTCVIGTVAGDLHDIGKNLVAMMLESVGFNVVDLGVDVSAE
KFVDAVRENDNVKIVACSGLLTTTMPAMKETVQSLKNSGLTGFKVIVGGAPVSQAMADEIGADGFAPDAGGAAV
KAKELAHAVD*

MT-L5-CP (dhaf-MT-L5-dhaf-CP):

DNA sequence

catatg**ATG**CTGACCATTAAACAGAATCTGCTGGAAACCATTCGTGGTGGTAATCCG-
GATCGTTTTGTTAATCAGTATGAGTTCATGGATATCATCCTGGAAAATCCGGTTGATTTTATGGCACCGCCTGG
TGGTAGCATTAAAACCGGTTGGGGTATTACCTTTCGTTGGCCTGAAGGTCAGATTGGTCAGTTTCCGGTTCATG
ATGAAGAACACAAAGTGCTGAAAGACATTACCAAATGGCGTGAACAGGTTAAAGCACCGGAAATTCCGGATA
CCGATGAAGCATGGGCAGCAGCAGTTGAACATGCAAATAGCATTGATCGCAACGAGAAATATGTTACCGCATT
TGTTGCACCGGGTGTTTTTGAAATGTGTCATCATCTGATGAGCATGGAAGATGCACTGATGGCACTGTATGAA
GAACCTGAACTGATGCATGAACTGATCGATTATCTGACCGAATATGAACTGAAACTGGCCGAAATTATCTGTC
GTCGTCTGAAACCGGATGCACTGTTTCATCACGATGATTGGGGTAGCCAGAAAAGCAGCTTTATTAGTCCGGC
AATGTTCGAAGAATTTTATCTGCCTGCCTACAAAAAAATCTATAGCTTCTATAAACAGAACGGCGTTGAACTG
ATTGTGCATCATAGCGATTGTTATGCAGCAAATCTGGTGCCGTTTATGATTGAAATGGGCATTGATATTTGGCA
GGGTGTTATGACCACCAATAATACACCGGAACTGATTAAACAGTACGGTGGCAAAATTAGCTTTATGGGCGAT
ATTGATAGCGGTGTTGTTGATTTTCCGACCTGGACCCGTGAAATTGCAGCACGTGAAGCAGAACGTGCATGTA
CCAATTGTGGTAAACATTATTTCATTCCGTGTCTGACCCAGGGTCTGGGTTTTAGCAGCTTTCCGGGTGTGTAT
GATTGTGTTAGCGAAGAAATTGATAAACTGAGCAAAAAAATGTTCGTCGACGAATTCGGTGGCGGTGGCTCGA
AGCTTATGAGCAAAATCGCCGAAGTTAAAGCAATGGTTGAAGCAGGTAAAGCAAAACTGGTTCCGGGTCTGG
TTCAAGAGGCACTGGATGCAGGTAATGCAGCCGGTGATATTCTGGCAGGTATGATTGATAGCATGGGTGTTGT
TGGTGATAAATTCAGTGCCGGTGAACTGTTTGTTCCGGAAATGCTGATGGCAGCAAAAGCAATGAGCAAAGGT
GTTGATGTTCTGAAACCGCATCTGACCGGTGAAAGCGCAACCAGCCTGGGCACCTGTGTTATTGGCACCGTTG
CCGGTGATCTGCATGATATTGGTAAAAATCTGGTTGCCATGATGCTGGAAAGCGTTGGTTTTAATGTTGTTGAT
CTGGGTGTGGATGTTAGCGCAGAAAAATTTGTTGATGCCGTGCGCGAAAATGACAACGTTAAAATTGTTGCAT

GTAGCGGTCTGCTGACCACCACCATGCCTGCAATGAAAGAAACCGTTCAGAGCCTGAAAAATTCAGGTCTGAC
CGGCTTTAAAGTTATTGTTGGTGGTGCACCGGTTAGCCAGGCAATGGCAGATGAAATTGGTGCAGATGGTTTT
GCACCGGATGCCGGTGGTGCAGCAGTTAAAGCCAAAGAACTGGCACATGCAGTCGAC**TAA**<u>ctcgag</u>

<u>catatg</u>= *NdeI*, <u>ctcgag</u>= *XhoI*, <u>GAATTC</u>= *EcoRI*, <u>AAGCTT</u>= *HindIII*

<u>Amino acid sequence</u>

MLTIKQNLLETIRGGNPDRFVNQYEFMDIILENPVDFMAPPGGSIKTGWGITFRWPEGQIGQFPVHDEEHKVLKDITK
WREQVKAPEIPDTDEAWAAAVEHANSIDRNEKYVTAFVAPGVFEMCHHLMSMEDALMALYEEPELMHELIDYLT
EYELKLAEIICRRLKPDALFHHDDWGSQKSSFISPAMFEEFYLPAYKKIYSFYKQNGVELIVHHSDCYAANLVPFMIE
MGIDIWQGVMTTNNTPELIKQYGGKISFMGDIDSGVVDFPTWTREIAAREAERACTNCGKHYFIPCLTQGLGFSSFP
GVYDCVSEEIDKLSKKMFVDEFGGGGSKLMSKIAEVKAMVEAGKAKLVPGLVQEALDAGNAAGDILAGMIDSMG
VVGDKFSAGELFVPEMLMAAKAMSKGVDVLKPHLTGESATSLGTCVIGTVAGDLHDIGKNLVAMMLESVGFNVV
DLGVDVSAEKFVDAVRENDNVKIVACSGLLTTTMPAMKETVQSLKNSGLTGFKVIVGGAPVSQAMADEIGADGFA
PDAGGAAVKAKELAHAVD*

MT-L10-CP (dhaf-MT-L5-dhaf-CP):

<u>DNA sequence</u>

<u>catatg</u>**ATG**CTGACCATTAAACAGAATCTGCTGGAAACCATTCGTGGTGGTAATCCG-
GATCGTTTTGTTAATCAGTATGAGTTCATGGATATCATCCTGGAAAATCCGGTTGATTTTATGGCACCGCCTGG
TGGTAGCATTAAAACCGGTTGGGGTATTACCTTTCGTTGGCCTGAAGGTCAGATTGGTCAGTTTCCGGTTCATG
ATGAAGAACACAAAGTGCTGAAAGACATTACCAAATGGCGTGAACAGGTTAAAGCACCGGAAATTCCGGATA
CCGATGAAGCATGGGCAGCAGCAGTTGAACATGCAAATAGCATTGATCGCAACGAGAAATATGTTACCGCATT
TGTTGCACCGGGTGTTTTTGAAATGTGTCATCATCTGATGAGCATGGAAGATGCACTGATGGCACTGTATGAA
GAACCTGAACTGATGCATGAACTGATCGATTATCTGACCGAATATGAACTGAAACTGGCCGAAATTATCTGTC
GTCGTCTGAAACCGGATGCACTGTTTCATCACGATGATTGGGGTAGCCAGAAAAGCAGCTTTATTAGTCCGGC
AATGTTCGAAGAATTTTATCTGCCTGCCTACAAAAAAATCTATAGCTTCTATAAACAGAACGGCGTTGAACTG
ATTGTGCATCATAGCGATTGTTATGCAGCAAATCTGGTGCCGTTTATGATTGAAATGGGCATTGATATTTGGCA
GGGTGTTATGACCACCAATAATACACCGGAACTGATTAAACAGTACGGTGGCAAAATTAGCTTTATGGGCGAT
ATTGATAGCGGTGTTGTTGATTTTCCGACCTGGACCCGTGAAATTGCAGCACGTGAAGCAGAACGTGCATGTA
CCAATTGTGGTAAACATTATTTCATTCCGTGTCTGACCCAGGGTCTGGGTTTTAGCAGCTTTCCGGGTGTGTAT
GATTGTGTTAGCGAAGAAATTGATAAACTGAGCAAAAAAATGTTCGTCGAC<u>GAATTC</u>GGTGGCGGTGGCTCGG
GCGGTGGTGGGTCG<u>AAGCTT</u>ATGAGCAAAATCGCCGAAGTTAAAGCAATGGTTGAAGCAGGTAAAGCAAAAC
TGGTTCCGGGTCTGGTTCAAGAGGCACTGGATGCAGGTAATGCAGCCGGTGATATTCTGGCAGGTATGATTGA
TAGCATGGGTGTTGTTGGTGATAAATTCAGTGCCGGTGAACTGTTTGTTCCGGAAATGCTGATGGCAGCAAAA
GCAATGAGCAAAGGTGTTGATGTTCTGAAACCGCATCTGACCGGTGAAAGCGCAACCAGCCTGGGCACCTGTG
TTATTGGCACCGTTGCCGGTGATCTGCATGATATTGGTAAAAATCTGGTTGCCATGATGCTGGAAAGCGTTGGT
TTTAATGTTGTTGATCTGGGTGTGGATGTTAGCGCAGAAAAATTTGTTGATGCCGTGCGCGAAAATGACAACG
TTAAAATTGTTGCATGTAGCGGTCTGCTGACCACCACCATGCCTGCAATGAAAGAAACCGTTCAGAGCCTGAA
AAATTCAGGTCTGACCGGCTTTAAAGTTATTGTTGGTGGTGCACCGGTTAGCCAGGCAATGGCAGATGAAATT
GGTGCAGATGGTTTTGCACCGGATGCCGGTGGTGCAGCAGTTAAAGCCAAAGAACTGGCACATGCAGTCGAC**T
AA**<u>ctcgag</u>

<u>catatg</u>= *NdeI*, <u>ctcgag</u>= *XhoI*, <u>GAATTC</u>= *EcoRI*, <u>AAGCTT</u>= *HindIII*

<u>Amino acid sequence</u>

MLTIKQNLLETIRGGNPDRFVNQYEFMDIILENPVDFMAPPGGSIKTGWGITFRWPEGQIGQFPVHDEEHKVLKDITK
WREQVKAPEIPDTDEAWAAAVEHANSIDRNEKYVTAFVAPGVFEMCHHLMSMEDALMALYEEPELMHELIDYLT
EYELKLAEIICRRLKPDALFHHDDWGSQKSSFISPAMFEEFYLPAYKKIYSFYKQNGVELIVHHSDCYAANLVPFMIE
MGIDIWQGVMTTNNTPELIKQYGGKISFMGDIDSGVVDFPTWTREIAAREAERACTNCGKHYFIPCLTQGLGFSSFP

GVYDCVSEEIDKLSKKMFVDEFGGGGSGGGGSKLMSKIAEVKAMVEAGKAKLVPGLVQEALDAGNAAGDILAGM
IDSMGVVGDKFSAGELFVPEMLMAAKAMSKGVDVLKPHLTGESATSLGTCVIGTVAGDLHDIGKNLVAMMLESV
GFNVVDLGVDVSAEKFVDAVRENDNVKIVACSGLLTTTMPAMKETVQSLKNSGLTGFKVIVGGAPVSQAMADEIG
ADGFAPDAGGAAVKAKELAHAVD*

MT-L15-CP (dhaf-MT-L5-dhaf-CP):

DNA sequence

catatg**ATG**CTGACCATTAAACAGAATCTGCTGGAAACCATTCGTGGTGGTAATCCG-
GATCGTTTTGTTAATCAGTATGAGTTCATGGATATCATCCTGGAAAATCCGGTTGATTTTATGGCACCGCCTGG
TGGTAGCATTAAAACCGGTTGGGGTATTACCTTTCGTTGGCCTGAAGGTCAGATTGGTCAGTTTCCGGTTCATG
ATGAAGAACACAAAGTGCTGAAAGACATTACCAAATGGCGTGAACAGGTTAAAGCACCGGAAATTCCGGATA
CCGATGAAGCATGGGCAGCAGCAGTTGAACATGCAAATAGCATTGATCGCAACGAGAAATATGTTACCGCATT
TGTTGCACCGGGTGTTTTTGAAATGTGTCATCATCTGATGAGCATGGAAGATGCACTGATGGCACTGTATGAA
GAACCTGAACTGATGCATGAACTGATCGATTATCTGACCGAATATGAACTGAAACTGGCCGAAATTATCTGTC
GTCGTCTGAAACCGGATGCACTGTTTCATCACGATGATTGGGGTAGCCAGAAAAGCAGCTTTATTAGTCCGGC
AATGTTCGAAGAATTTTATCTGCCTGCCTACAAAAAAATCTATAGCTTCTATAAACAGAACGGCGTTGAACTG
ATTGTGCATCATAGCGATTGTTATGCAGCAAATCTGGTGCCGTTTATGATTGAAATGGGCATTGATATTTGGCA
GGGTGTTATGACCACCAATAATACACCGGAACTGATTAAACAGTACGGTGGCAAAATTAGCTTTATGGGCGAT
ATTGATAGCGGTGTTGTTGATTTTCCGACCTGGACCCGTGAAATTGCAGCACGTGAAGCAGAACGTGCATGTA
CCAATTGTGGTAAACATTATTTCATTCCGTGTCTGACCCAGGGTCTGGGTTTTAGCAGCTTTCCGGGTGTGTAT
GATTGTGTTAGCGAAGAAATTGATAAACTGAGCAAAAAAATGTTCGTCGACGAATTCGGTGGCGGTGGCTCGG
GCGGTGGTGGGTCGGGTGGCGGCGGATCCAAGCTTATGAGCAAAATCGCCGAAGTTAAAGCAATGGTTGAAG
CAGGTAAAGCAAAACTGGTTCCGGGTCTGGTTCAAGAGGCACTGGATGCAGGTAATGCAGCCGGTGATATTCT
GGCAGGTATGATTGATAGCATGGGTGTTGTTGGTGATAAATTCAGTGCCGGTGAACTGTTTGTTCCGGAAATG
CTGATGGCAGCAAAAGCAATGAGCAAAGGTGTTGATGTTCTGAAACCGCATCTGACCGGTGAAAGCGCAACC
AGCCTGGGCACCTGTGTTATTGGCACCGTTGCCGGTGATCTGCATGATATTGGTAAAAATCTGGTTGCCATGAT
GCTGGAAAGCGTTGGTTTTAATGTTGTTGATCTGGGTGTGGATGTTAGCGCAGAAAAATTTGTTGATGCCGTGC
GCGAAAATGACAACGTTAAAATTGTTGCATGTAGCGGTCTGCTGACCACCACCATGCCTGCAATGAAAGAAAC
CGTTCAGAGCCTGAAAAATTCAGGTCTGACCGGCTTTAAAGTTATTGTTGGTGGTGCACCGGTTAGCCAGGCA
ATGGCAGATGAAATTGGTGCAGATGGTTTTGCACCGGATGCCGGTGGTGCAGCAGTTAAAGCCAAAGAACTG
GCACATGCAGTCGAC**TAA**ctcgag

catatg= *NdeI*, ctcgag= *XhoI*, GAATTC= *EcoRI*, AAGCTT= *HindIII*

Amino acid sequence

MLTIKQNLLETIRGGNPDRFVNQYEFMDIILENPVDFMAPPGGSIKTGWGITFRWPEGQIGQFPVHDEEHKVLKDITK
WREQVKAPEIPDTDEAWAAAVEHANSIDRNEKYVTAFVAPGVFEMCHHLMSMEDALMALYEEPELMHELIDYLT
EYELKLAEIICRRLKPDALFHHDDWGSQKSSFISPAMFEEFYLPAYKKIYSFYKQNGVELIVHHSDCYAANLVPFMIE
MGIDIWQGVMTTNNTPELIKQYGGKISFMGDIDSGVVDFPTWTREIAAREAERACTNCGKHYFIPCLTQGLGFSSFP
GVYDCVSEEIDKLSKKMFVDEFGGGGSGGGGSGGGGSKLMSKIAEVKAMVEAGKAKLVPGLVQEALDAGNAAG
DILAGMIDSMGVVGDKFSAGELFVPEMLMAAKAMSKGVDVLKPHLTGESATSLGTCVIGTVAGDLHDIGKNLVA
MMLESVGFNVVDLGVDVSAEKFVDAVRENDNVKIVACSGLLTTTMPAMKETVQSLKNSGLTGFKVIVGGAPVSQ
AMADEIGADGFAPDAGGAAVKAKELAHAVD*

3. Methods

3.1 DNA sequence alignment of cmuA with *dhaf*-MT and *dhaf*-CP

The pairwise sequence alignment of the natural fusion enzyme cmuA (*Hyphomicrobium chloromethanicum*) was used to determine the orientation (*N*-terminal or C-terminal) in which the *dhaf*-MT and *dhaf*-CP should be placed to each other for the cloning of the new recombinant fusion enzymes. For the two sequence alignments the amino sequence of cmuA was aligned against the sequences of *dhaf*-MT and *dhaf*-CP with the online-available software EMBOSS Needle (https://www.ebi.ac.uk/Tools/psa/emboss_needle/). Regarding the alignment, cmuA had the highest score with N-terminal *dhaf*-MT and C-terminal *dhaf*-CP (22% sequence identity) compared to the other orientation (8% sequence identity).

<u>Alignment of cmuA (template) with N-terminal *dhaf*-MT and C-terminal *dhaf*-CP</u>

```
#=======================================
# Aligned_sequences: 2
# 1: cmuA (EMBOSS_001)
# 2: dhaf4610-dhaf4611
# Matrix: EBLOSUM62
# Gap_penalty: 10.0
# Extend_penalty: 0.5
#
# Length: 675
# Identity:     147/675 (21.8%)
# Similarity:   250/675 (37.0%)
# Gaps:         197/675 (29.2%)
# Score: 316.0
#=======================================

EMBOSS_001         1 MTQVPKMTSRERLFAAVTMQTLPDQVPCVPLLMTRGIREGGITVDQALRD     50
                                                             .:|:.|.|  
EMBOSS_001         1 ----------------------------------------MLTIKQNL--      8

EMBOSS_001        51 GEASAHAKIKALKKFGG------------DVII---------PGTDLFTP     79
                             ::.::  ||            |:|:         ||..:.| 
EMBOSS_001         9 --------LETIR--GGNPDRFVNQYEFMDIILENPVDFMAPPGGSIKT-     47

EMBOSS_001        80 VECVEGCELDYLPYAQPSLVKHPTPTKEAFYRYKEKYLREGFKPSERV--    127
                          |..:.: .:.:..:.:.|...:|      .|.|::..|..|:|  
EMBOSS_001        48 -----GWGITF-RWPEGQIGQFPVHDEE------HKVLKDITKWREQVKA     85

EMBOSS_001       128 -----------LQIQRARTMIARRKDTHAMPTPVGGPITRAQLMTGSSEF    166
                                ..::.|.::....|...|...|  |.......:....:.
EMBOSS_001        86 PEIPDTDEAWAAAVEHANSIDRNEKYVTAFVAP--GVFEMCHHLMSMEDA    133

EMBOSS_001       167 LSYISDDPDYAKE----VTELALDIVKNVCRMMFEAGIDVCNILDPFNS-    211
                     |..:.::|:...|    :||..|.:.:.:||.:..   |.....|.:.| 
EMBOSS_001       134 LMALYEEPELMHELIDYLTEYELKLAEIICRRLKP---DALFHHDDWGSQ    180

EMBOSS_001       212 -SDILPPDTYREFGLPYQKRLFAYIKEIGGIGFTHTCTFTQPIWRDIANN    260
                      |..:.|..:.||.||..|:::::.|:.|.....|             ::
EMBOSS_001       181 KSSFISPAMFEEFYLPAYKKIYSFYKQNGVELIVH-------------HS    217

EMBOSS_001       261 GCFNFN--------G-DMYPGM------DHAKRAIGGQISLMGTLSPFST    295
                     .|:..|        | |::.|:      ....:..||:||.||.:.....
EMBOSS_001       218 DCYAANLVPFMIEMGIDIWQGVMTTNNTPELIKQYGGKISFMGDIDSGVV    267

EMBOSS_001       296 FMHGWTTDVANKVKKLAAEVGYNGGLIVMPGCDIDWTIPDENLKAMIETC    345
                     ....||.::|.:..:.|.           ..|...:.||          |
EMBOSS_001       268 DFPTWTREIAAREAERAC-----------TNCGKHYFIP----------C    296

EMBOSS_001       346 ASIKYPMDVAALGDLSNVYLAGHPKHPGKRAPSTAGDTD-VAEA--KTHH    392
                     .:                        .|....|..|..| |:|.  |...
EMBOSS_001       297 LT------------------------QGLGFSSFPGVYDCVSEEIDKLSK    322

EMBOSS_001       393 KELTPQQEVNEKLVEAIMEYDGDKAIE-WVKKGLERGMTAQDIVFDGLSL    441
                     |......::.|  |:|::|....|.:. .|::.|:.|..|.||: .|:..
EMBOSS_001       323 KMFVDMSKIAE--VKAMVEAGKAKLVPGLVQEALDAGNAAGDIL-AGMID    369
```

```
EMBOSS_001       442 GMKVVGDMYERNERFVTDMLKAAKTMDKAMPILTPLLEQAGGDGGPTGTV    491
                     .|.||||.:...|.||.:||.|||.|.|.:.:|.|.|  .|......||.
EMBOSS_001       370 SMGVVGDKFSAGELFVPEMLMAAKAMSKGVDVLKPHL--TGESATSLGTC    417

EMBOSS_001       492 VVGLVRGNTQDIGKNLVCLMLKANGFKVIDLGKNVKPEQFIESA-EKENA    540
                     |:|.|.|:..|||||||.:||::.||.|:|||.:|..|:|:::. |.:|.
EMBOSS_001       418 VIGTVAGDLHDIGKNLVAMMLESVGFNVVDLGVDVSAEKFVDAVRENDNV    467

EMBOSS_001       541 VAIGMS-VMTNSSTVYVEKVKELLDKAGKGDKYLLMCGGAAANKGVADKM    589
                     ..:..| ::|.:.....|.|:.|.:....|.|.::  |||..::.:||::
EMBOSS_001       468 KIVACSGLLTTTMPAMKETVQSLKNSGLTGFKVIV--GGAPVSQAMADEI    515

EMBOSS_001       590 GVKYGLDANAAVSLVKDHLQAAALE    614
                     |.. |...:|..:.||....|.|::
EMBOSS_001       516 GAD-GFAPDAGGAAVKAKELAHAVD    539
```

Alignment of cmuA (template) with N-terminal *dhaf*-CP and C-terminal *dhaf*-MT

```
#=======================================
# Aligned_sequences: 2
# 1: cmuA (EMBOSS_001)
# 2: dhaf4611-dhaf4610
# Matrix: EBLOSUM62
# Gap_penalty: 10.0
# Extend_penalty: 0.5
#
# Length: 944
# Identity:      76/944 (8.1%)
# Similarity:   120/944 (12.7%)
# Gaps:         735/944 (77.9%)
# Score: 267.5
#=======================================

EMBOSS_001         1 MTQVPKMTSRERLFAAVTMQTLPDQVPCVPLLMTRGIREGGITVDQALRD     50
EMBOSS_001         1 --------------------------------------------------      0

EMBOSS_001        51 GEASAHAKIKALKKFGGDVIIPGTDLFTPVECVEGCELDYLPYAQPSLVK    100
EMBOSS_001         1 --------------------------------------------------      0

EMBOSS_001       101 HPTPTKEAFYRYKEKYLREGFKPSERVLQIQRARTMIARRKDTHAMPTPV    150
EMBOSS_001         1 --------------------------------------------------      0

EMBOSS_001       151 GGPITRAQLMTGSSEFLSYISDDPDYAKEVTELALDIVKNVCRMMFEAGI    200
EMBOSS_001         1 --------------------------------------------------      0

EMBOSS_001       201 DVCNILDPFNSSDILPPDTYREFGLPYQKRLFAYIKEIGGIGFTHTCTFT    250
EMBOSS_001         1 --------------------------------------------------      0
EMBOSS_001       251 QPIWRDIANNGCFNFNGDMYPGMDHAKRAIGGQISLMGTLSPFSTFMHGW    300
EMBOSS_001         1 --------------------------------------------------      0

EMBOSS_001       301 TTDVANKVKKLAAEVGYNGGLIVMPGCDIDWTIPDENLKAMIETCASIKY    350
EMBOSS_001         1 --------------------------------------------------      0

EMBOSS_001       351 PMDVAALGDLSNVYLAGHPKHPGKRAPSTAGDTDVAEAKTHHKELTPQQE    400
                                                                    ..:
EMBOSS_001         1 -----------------------------------------------MSK      3

EMBOSS_001       401 VNEKLVEAIMEYDGDKAIE-WVKKGLERGMTAQDIVFDGLSLGMKVVGDM    449
                     :.|  |:|::|....|.:. .|::.|:.|..|.||: .|:...|.||||.
EMBOSS_001         4 IAE--VKAMVEAGKAKLVPGLVQEALDAGNAAGDIL-AGMIDSMGVVGDK     50

EMBOSS_001       450 YERNERFVTDMLKAAKTMDKAMPILTPLLEQAGGDGGPTGTVVVGLVRGN    499
                     :...|.||.:||.|||.|.|.:.:|.|.|  .|......||.|:|.|.|:
EMBOSS_001        51 FSAGELFVPEMLMAAKAMSKGVDVLKPHL--TGESATSLGTCVIGTVAGD     98

EMBOSS_001       500 TQDIGKNLVCLMLKANGFKVIDLGKNVKPEQFIESA-EKENAVAIGMS-V    547
                     ..|||||||.:||::.||.|:|||.:|..|:|:::. |.:|...:..| :
EMBOSS_001        99 LHDIGKNLVAMMLESVGFNVVDLGVDVSAEKFVDAVRENDNVKIVACSGL    148

EMBOSS_001       548 MTNSSTVYVEKVKELLDKAGKGDKYLLMCGGAAANKGVADKMGVKYGLDA    597
                     :|.:.....|.|:.|.:....|.|.::  |||..::.:||::|.. |...
EMBOSS_001       149 LTTTMPAMKETVQSLKNSGLTGFKVIV--GGAPVSQAMADEIGAD-GFAP    195
```

```
EMBOSS_001       598 NAAVSLVKDHLQAAALE---------------------------------    614
                     :|..:.||....|.|::
EMBOSS_001       196 DAGGAAVKAKELAHAVDMLTIKQNLLETIRGGNPDRFVNQYEFMDIILEN    245

EMBOSS_001       615 --------------------------------------------------    614
EMBOSS_001       246 PVDFMAPPGGSIKTGWGITFRWPEGQIGQFPVHDEEHKVLKDITKWREQV    295

EMBOSS_001       615 --------------------------------------------------    614
EMBOSS_001       296 KAPEIPDTDEAWAAAVEHANSIDRNEKYVTAFVAPGVFEMCHHLMSMEDA    345

EMBOSS_001       615 --------------------------------------------------    614
EMBOSS_001       346 LMALYEEPELMHELIDYLTEYELKLAEIICRRLKPDALFHHDDWGSQKSS    395

EMBOSS_001       615 --------------------------------------------------    614
EMBOSS_001       396 FISPAMFEEFYLPAYKKIYSFYKQNGVELIVHHSDCYAANLVPFMIEMGI    445

EMBOSS_001       615 --------------------------------------------------    614
EMBOSS_001       446 DIWQGVMTTNNTPELIKQYGGKISFMGDIDSGVVDFPTWTREIAAREAER    495

EMBOSS_001       615 --------------------------------------------    614
EMBOSS_001       496 ACTNCGKHYFIPCLTQGLGFSSFPGVYDCVSEEIDKLSKKMFVD    539
```

3.2 Protein expression of fusion enzymes and *dhaf*-MT and *dhaf*-CP

All recombinant fusion proteins as well as the separate proteins *dhaf*-MT and *dhaf*-CP were expressed in soluble form (**lanes 1-6**, Figure S10). The protein content for MT-CP (1.0 µg, 17.3 pmol, **lane 1**, Figure S10), MT-L5-CP (0.73 µg, 12.3 pmol, l**ane 2**, Figure S10), MT-L10-CP (0.7 µg, 11.7 pmol, **lane 3**, Figure S10), MT-L15-CP (0.59 µg, 9.8 pmol, Figure S10), *dhaf*-MT (0.53 µg, 14.3 pmol, **lane 5**, Figure S10) and *dhaf*-CP (0.45 µg, 21 pmol, **lane 6**, Figure S10) were calculated by densitometry (ImageJ). With this, the stoichiometric ratio of 1:1.5 (*dhaf*-MT/*dhaf*-CP) was determined as a positive control to compare the reactivity of the fusion proteins.

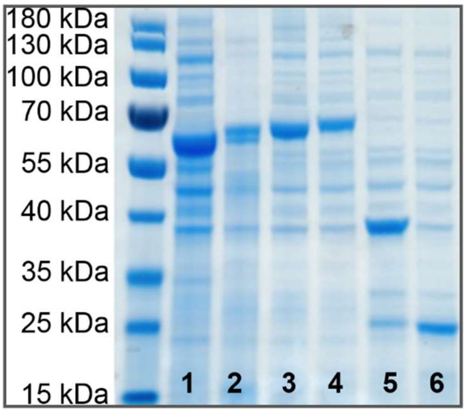

Figure S10. SDS-PAGE of fusion enzymes as well as *dhaf*-MT and *dhaf*-CP. Recombinant fusion enzymes: MT-CP (**lane 1**, 58.9 kDa), MT-L5-CP (**lane 2**, 59.8 kDa), MT-L10-CP (**lane 3**, 60.1 kDa) and MT-L15-CP (**lane 4**, 60.4 kDa). Proteins from *Desulfitobacterium dehafniense*: *dhaf*-MT (**lane 5**, 37.5 kDa) and *dhaf*-CP (**lane 6**, 21.4 kDa). All proteins were applied with 15 µg protein per lane.

3.3 Protein purification of MT-L5-CP

The fusion protein MT-L5-CP containing a *N*-terminal His-tag was successfully purified with IMAC (5 mL nickel column, Figure S11, left) and size exclusion chromatography (Superdex S75 column, Figure S11, right) yielding in high purity with the assigned protein band at 60 kDa.

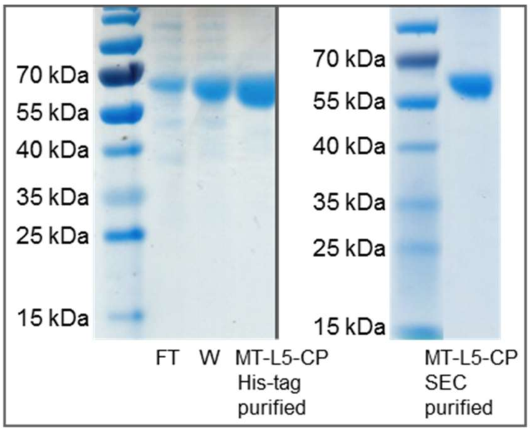

Figure S11. SDS-PAGE of the protein purification of the recombinant fusion protein MT-L5-CP. The carrier protein was successfully purified by ion-metal affinity chromatography (IMAC) and size-exclusion chromatography (SEC) according to Cytiva purification protocols leading to a pure protein band at 60 kDa. All SDS samples were applied with 10 µg protein.

3.4 Determination of the pure MT-L5-CP content in the CFE

First, the fusion protein MT-L5-CP was expressed in *E. coli* BL21(DE3) (0.5 mM IPTG at 25 °C for 24 h). Then, the protein concentration of the lyophilized cell-free extract (CFE) was measured by Bradford (68 mg/mL). After the purification, the concentration was measured again by Bradford and gave 12 mg/mL for pure MT-L5-CP. Thus, 68 mg/mL lyophilized cell-free extract contained 12 mg/mL pure MT-L5-CP. Therefore, 17.6% of the total CFE consisted out of the fusion enzyme MT-L5-CP.

3.5 Preparations of biocatalysts

3.5.1 *Holo*-CP and *holo*-fusion enzyme loading

The loading of the *dhaf*-CP or the fusion enzyme with its cofactor methylcobalamin hydrate was performed under inert atmosphere according to the optimised protocol in literature.[1] First the reconstitution buffer was prepared consisting of methylcobalamin (6 mM) and betaine (3 M) dissolved in Tris/HCl buffer (50 mM, pH 7, 2.5 mM DTT, 0.1 mM PMSF). The lyophilized *dhaf*-CP or fusion enzyme (*dhaf*-CP: 104 mg/mL CFE≡ 22 mg/mL pure protein;[2] fusion enzymes: 208 mg/mL CFE≡ 37 mg/mL) was mixed with the reconstitution buffer (1mL) and incubated for at least 2 h at 4°C. This incubation step was crucial for complete loading of the methylcobalamin onto the CP. The removal of salts and unbound cofactor was performed via a desalting step using a PD MidiTrap™ G-25 column (GE Healthcare) or PD 10™ G-25 column (GE Healthcare) according to the manufacturer's manual. During this step, the buffer was exchanged to MOPS buffer (50 mM, pH 6.5, 150 mM KCl). During this process, a red colored protein solution was obtained (*dhaf*-CP: 69.3 mg/mL CFE; fusion enzymes: 138.7 mg/mL CFE).

4. Analytics

4.1 TLC and Flash Column Chromatography

For the thin layer chromatography (TLC) Merck TLC silica gel 60 F254 plates were used. Each analyte had to be separated with the appropriate polar and nonpolar solvents (e.g. EtOAc/cyclohexane, 1/1) on TLC before the flash column chromatography was considered to isolate the substrates from their products. The purification was performed using silica gel 60 M (particle size 40-63 µm/230-400 mesh) from Macherey-Nagel as stationary phase.

4.2 HPLC-UV, HPLC-MS and preparative HPLC-UV

4.2.1 Method for HPLC-UV and HPLC-MS

The product formation and the conversion from biotransformations were analyzed by HPLC-UV (Agilent 1260 Infinity system equipped with a SPD-M20A diode array detector) on a reversed-phase achiral C18 column (Phenomenex Luna® C18, 100c, 250 x 4.6 mm, 5 mm). Injection volume was 10 µL each sample, and elution was done with a H_2O/MeCN (+0.1% TFA) gradient at 1 mL/min. *Method A*: 100% H_2O (2 min), 0-40% MeCN (13 min), 40-100% MeCN (5 min), 100% MeCN (2 min). *Method B*: 100% H_2O (2 min), 0-30% MeCN (13 min), 30-100% MeCN (5 min), 100% MeCN (2 min) The compounds were detected by UV-absorption at 280 nm (254 nm for **2e**) and the peaks were compared with commercially bought reference material for identification of the compounds and quantification of the conversion or product formation. All references were dissolved in MOPS buffer (50 mM, pH 6.5, 150 mM KCl, addition of 20 or 50% DMSO if necessary) and calibrations curves with concentrations from 1 to 50 mM were prepared (Table S6). The obtained slope of the linear correlation (k-value) was used for quantification of the appropriate analyte based on following equation:

$$c_{analyte} = \frac{integral\,[mAU]}{k\,[mAU]}[mM] \quad \textbf{(2)}$$

The molecular mass of each analyte was verified by HPLC-MS analysis, which was performed on an Agilent 6120 Quadrupole LC/MS system. Acetonitrile/water as eluent on a Kinetex column as stationary phase (2.6 µm C18 100 Å, LC column 50 x 4.6 mm).

Table S6. Retention time and k-value of each compound analysed by HPLC-UV.

Compound	t_R [min]	k-value [mAU/mM]
caffeic acid **2b**	13	157.2
ferulic acid *m*-**1b**	15.6	140.9
isoferulic acid *p*-**1b**	15.9	201.8
3,4-dihydroxybenzaldehyde **2e**	13.2[a]	159.6[a]
vanillin *m*-**1e**	15.9	479.2
isovanillin *p*-**1e**	15.7	449.5
3,4-dihydroxybenzyl alcohol **2f**	9.6	133.7
vanillyl alcohol *m*-**1f**	11.8	152.3
isovanillyl alcohol *p*-**1f**	12.4	138.4
catechol **2a**	13.3	118.3
guaiacol **1a**	17.7	119.8
4-methylcatechol **2c**	15.3	116.8
2-methoxy-5-methylphenol **1c**	19.8	120.9
caffeoyl alcohol **2f**	11.8[b]	245.6[b]

all standards were measured with HPLC Method A, λ= 280 nm
[a] standards were measured with HPLC Method A, λ= 262 nm
[b] standards were measured with HPLC Method B, λ= 280 nm

4.2.2 HPLC chromatograms

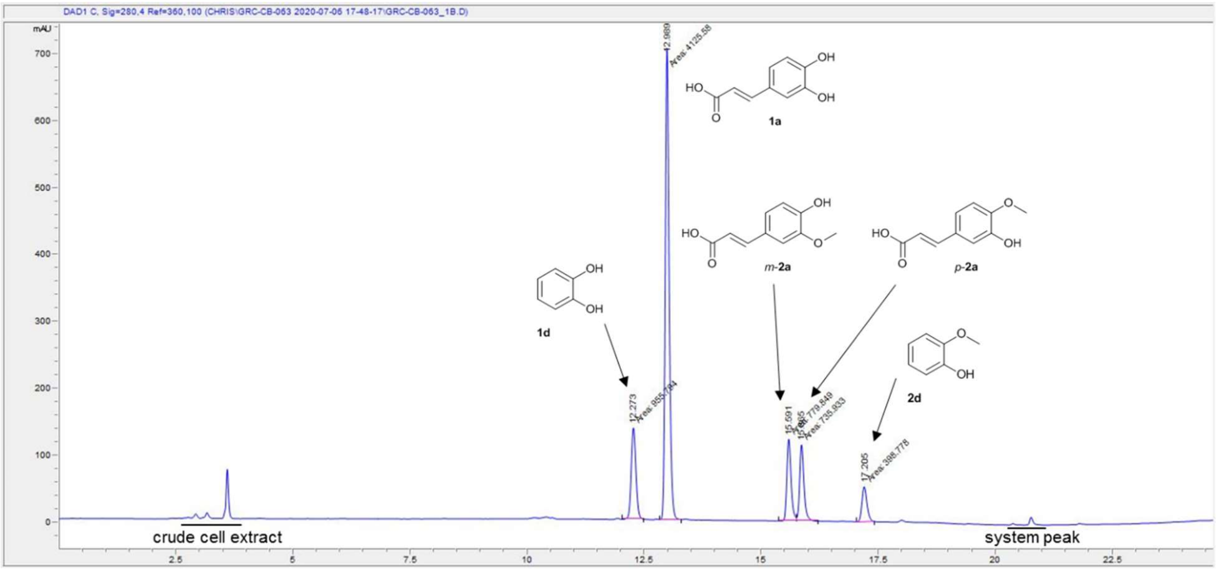

Figure S12. HPLC chromatogram of the demethylation of guaiacol 1a coupled to concomitant methylation of caffeic acid 2b. Reaction conditions: methyl donor **1a** (10 mM, 1.2 mg/mL), methyl acceptor **2b** (30 mM, 5.4 mg/mL), MT-L5-CP (60 mg/mL cell-free extract, ≡ 0.18 mM pure enzyme) with methylcobalamin (0.5 mg/mL, 0.37 mM) directly added to the reaction in MOPS buffer (50 mM, pH 6.5, 150 mM KCl) at 30 °C, 800 rpm in Eppendorf Thermomixer® (1.5 mL) for 3 h. All reactions were quenched by the addition of MeCN (60 vol. %) after 3 h and the conversions were analyzed via calibration curves of the corresponding reference compounds on HPLC-UV (*Method A*, 280 nm). The peak at 20.5 min appears independently and is assigned as system peak.

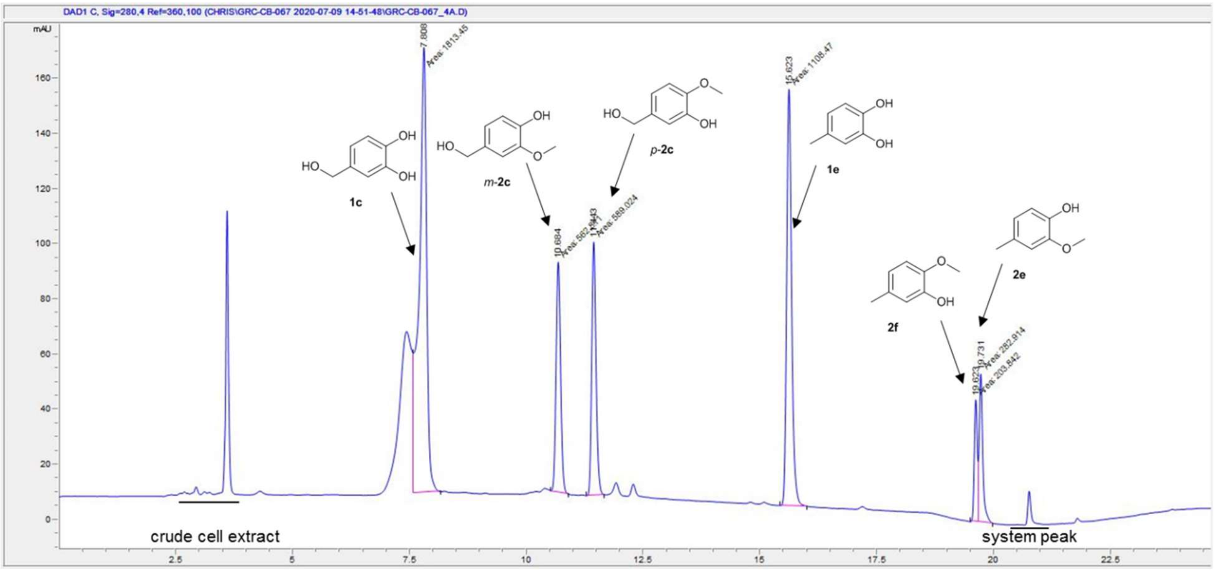

Figure S13. HPLC chromatogram of the demethylation of 2-methoxy-5-methylphenol 1c coupled to concomitant methylation of 3,4-dihydroxybenzyl alcohol 2f. Reaction conditions: methyl donor **1c** (10 mM, 1.4 mg/mL), methyl acceptor **2f** (30 mM, 4.2 mg/mL), MT-L5-CP (60 mg/mL cell-free extract, ≡ 0.18 mM pure enzyme) with methylcobalamin (0.5 mg/mL, 0.37 mM) directly added to the reaction in MOPS buffer (50 mM, pH 6.5, 150 mM KCl) at 30 °C, 800 rpm in Eppendorf Thermomixer® (1.5 mL) for 3 h. All reactions were quenched by the addition of MeCN (60 vol. %) after 3 h and the conversions were analysed via calibration curves of the corresponding reference compounds on HPLC-UV (*Method A*, 280 nm). The peak at 20.5 min appears independently and is assigned as system peak.

4.3 NMR spectroscopy

^{1}H-NMR spectra were recorded of unknown isolated products on AV II 300 MHz and AV III HD 300 MHz spectrometers from BRUKER PHYSICS in different deuterated co-solvents (e.g. $CDCl_3$). Furthermore, ^{13}C-NMR spectra were recorded on an AV-300 (75.5 MHz) spectrometer from Bruker Physics. All shifts are given in ppm and coupling constants (*J*) are given in Hz.

4.3.1 NMR – biotransformation of 2f and 1c (25 mL)

4-methylcatechol (**2c**) obtained from biocatalytic reaction after column chromatography (SiO_2).

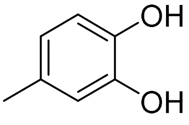

2c

^{1}H NMR (300 MHz, $CDCl_3$) δ 6.80 – 6.66 (m, 2H), 6.61 (d, *J* = 8.0 Hz, 1H), 5.23 (s, 2H), 2.24 (s, 3H).
^{13}C NMR (75 MHz, $CDCl_3$) δ 143.84, 141.57, 131.57, 121.96, 116.75, 115.81, 21.27.

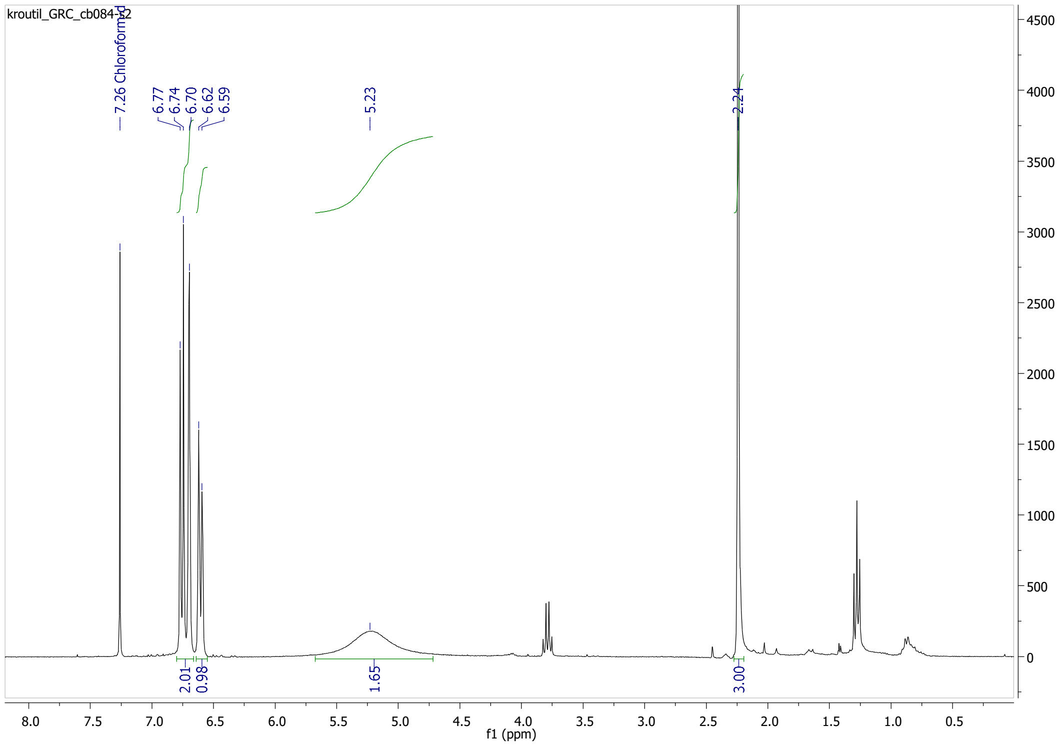

Figure S14. ^{1}H NMR spectra of 4-methylcatechol (**2c**) obtained from biocatalytic reaction after column chromatography (SiO_2). Additional signals refer to the cosolvents ethyl acetate (δ 2.05) and cyclohexane (δ 1.43).

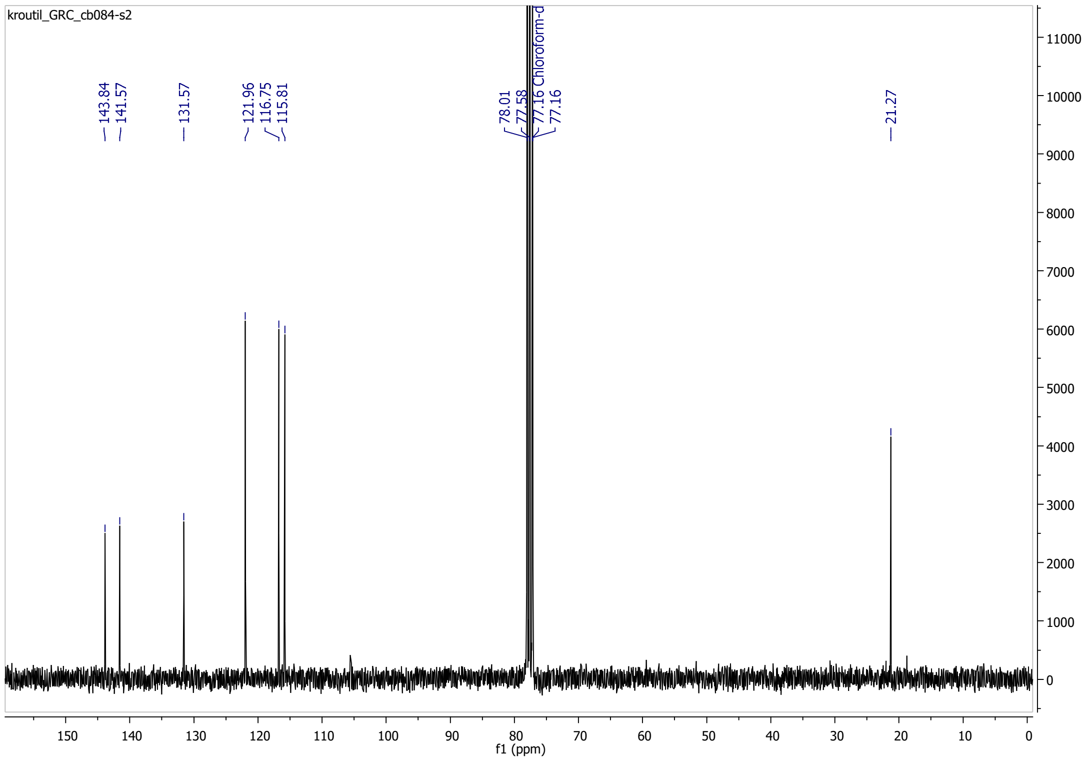

Figure S15. ^{13}C NMR spectra of 4-methylcatechol (**2c**) obtained from biocatalytic reaction after column chromatography (SiO_2).

Reaction mixture of vanillyl alcohol (*m*-**1f**) and isovanillyl alcohol (*p*-**1f**) isolated column chromatography (SiO_2):

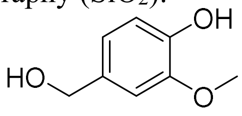

m-**1f**

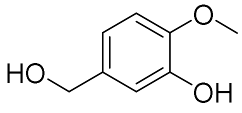

p-**1f**

^{1}H NMR (300 MHz, DMSO-d$_6$) δ 8.85 (s, 1H), 8.77 (s, 1H), 6.89 – 6.79 (m, 1H), 6.77 – 6.63 (m, 3H), 4.98 (s, 1H), 4.36 (s, 1H), 4.33 (s, 2H), 3.75 (s, 1H), 3.73 (s, 3H).

^{13}C NMR (75 MHz, DMSO-d$_6$) δ 147.3, 146.5, 146.3, 145.3, 135.3, 133.5, 119.1, 117.2, 115.0, 114.2, 111.9, 111.0, 63.0, 62.7, 55.7, 55.5.

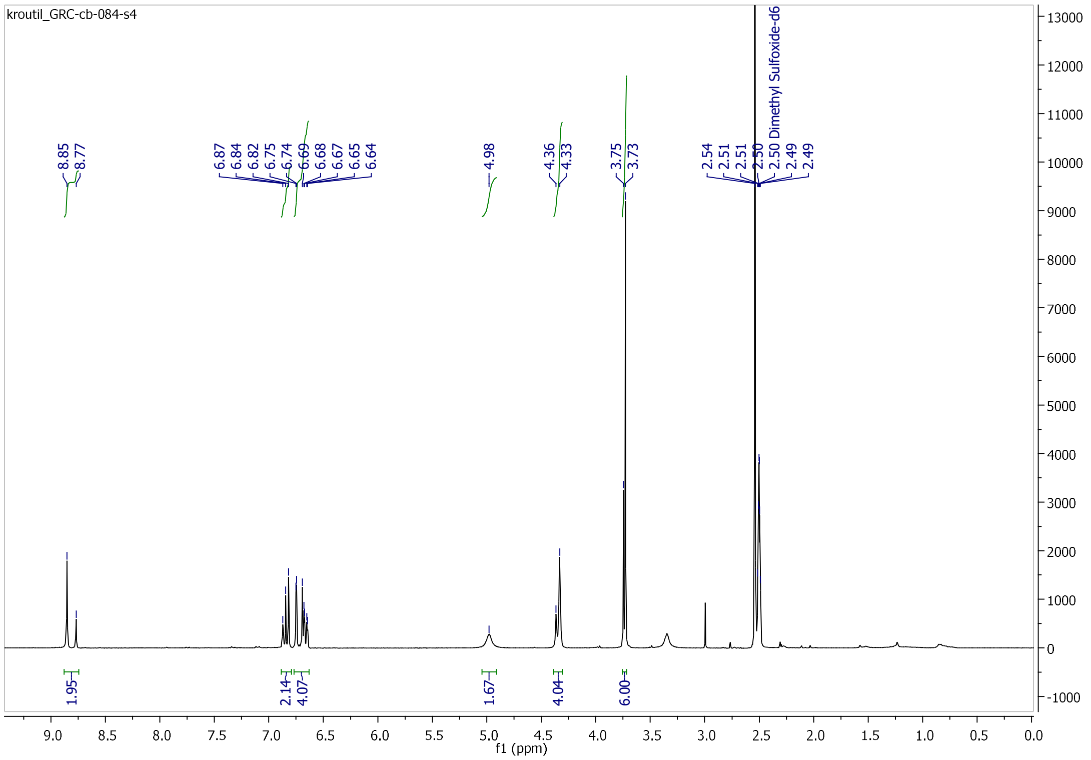

Figure S16. ^{1}H NMR spectra of vanillyl alcohol (*m*-**1f**) and isovanillyl alcohol (*p*-**1f**) obtained from biocatalytic reaction after column chromatography (SiO_2).

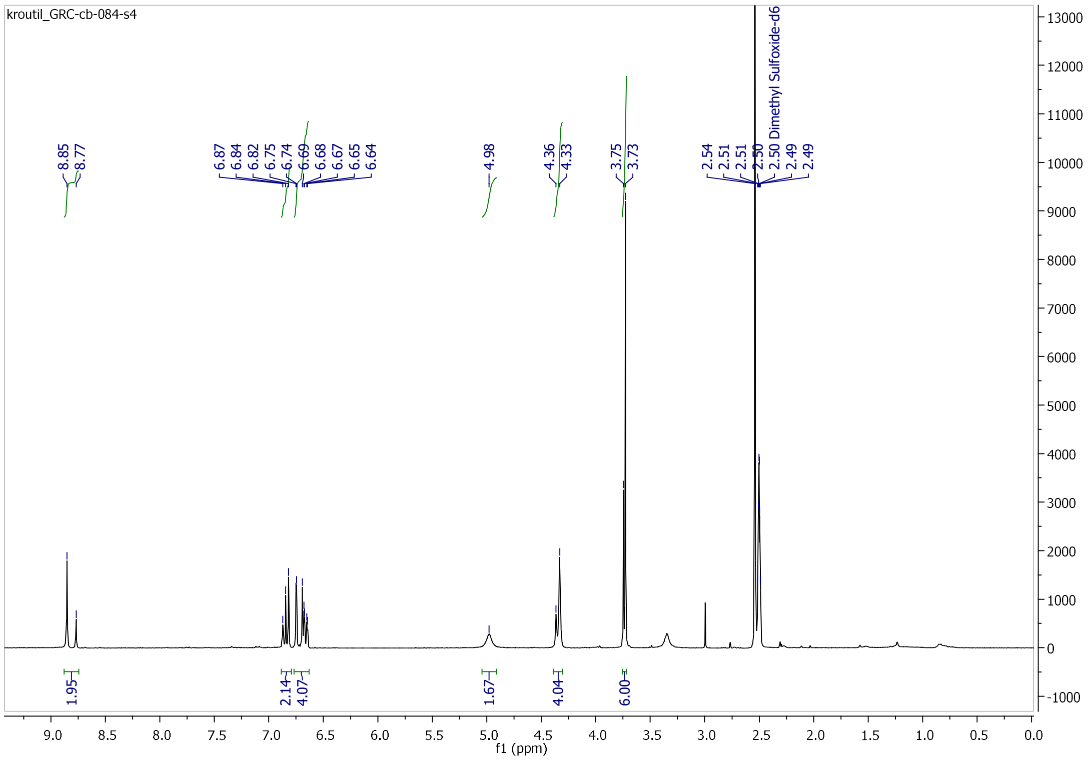

Figure S17. ^{1}H NMR spectra of vanillyl alcohol (*m*-**1f**) and isovanillyl alcohol (*p*-**1f**) obtained from biocatalytic reaction after column chromatography (SiO_2).

3,4-dihydroxybenzyl alcohol (**2f**) isolated column chromatography (SiO_2):

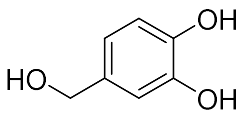

2f

^{1}H NMR (300 MHz, DMSO-d_6) δ 8.79 (s, 1H), 8.68 (s, 1H), 6.68 (dd, *J* = 17.9, 4.9 Hz, 2H), 6.54 (dd, *J* = 8.0, 2.0 Hz, 1H), 4.91 (t, *J* = 5.7 Hz, 1H), 4.29 (d, *J* = 5.7 Hz, 2H).
^{13}C NMR (75 MHz, DMSO-d_6) δ 144.97, 144.04, 133.56, 117.62, 115.16, 114.47, 62.95.

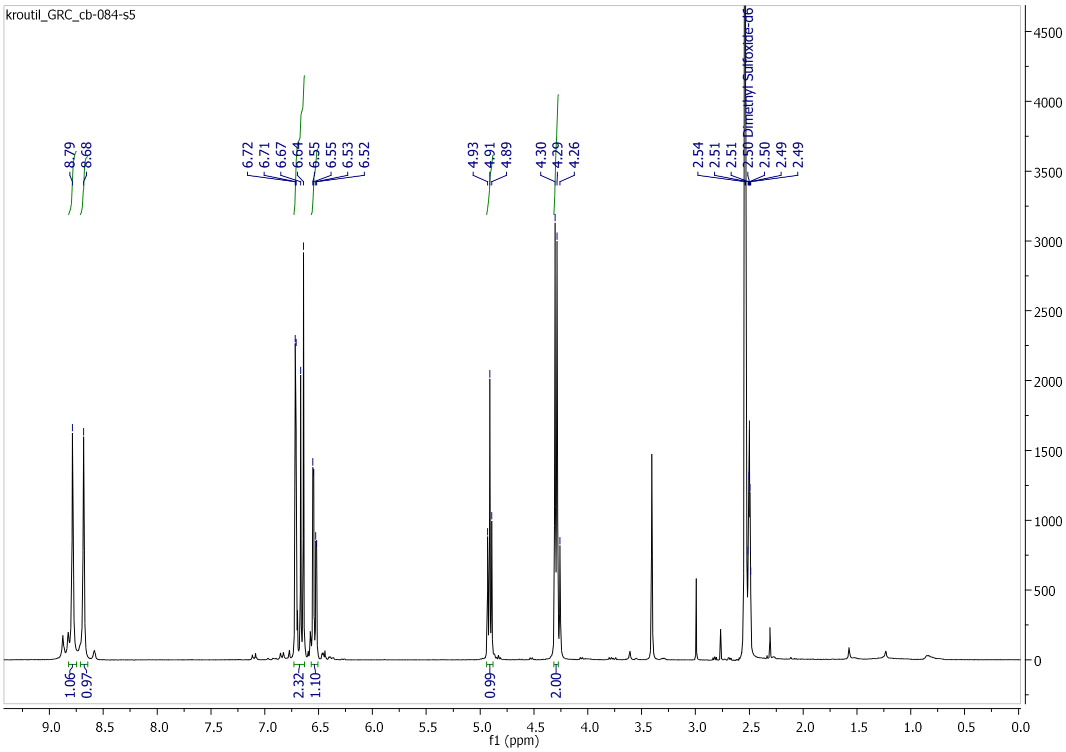

Figure S18. ^{1}H NMR spectra of 3,4-dihydroxybenzyl alcohol (**2f**) obtained from biocatalytic reaction after column chromatography (SiO_2).

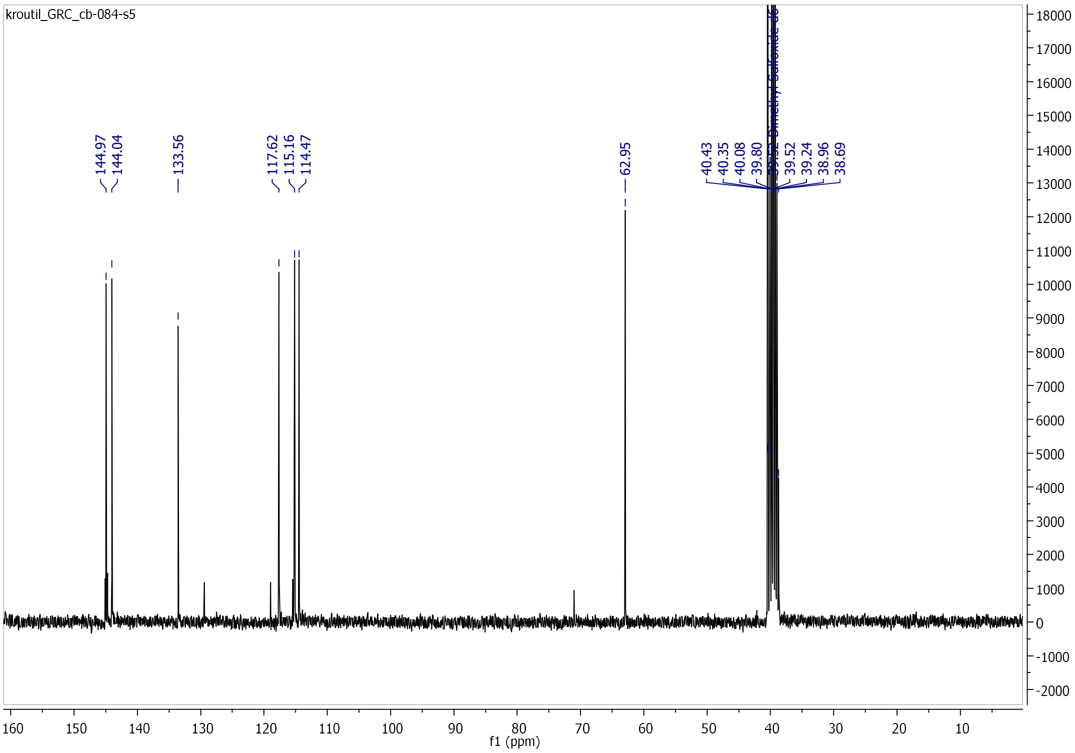

Figure S19. ^{13}C NMR spectra of 3,4-dihydroxybenzyl alcohol (**2f**) obtained from biocatalytic reaction after column chromatography (SiO_2).

4.3.2 NMRs – biotransformation of 1d and 2a (25 mL)

Catechol (**2a**) isolated column chromatography (SiO_2):

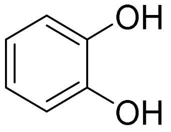

2a

^{1}H NMR (300 MHz, $CDCl_3$) δ 6.92 – 6.77 (m, 4H), 5.20 (s, 2H).
^{13}C NMR (75 MHz, $CDCl_3$) δ 143.6, 121.4, 115.7.

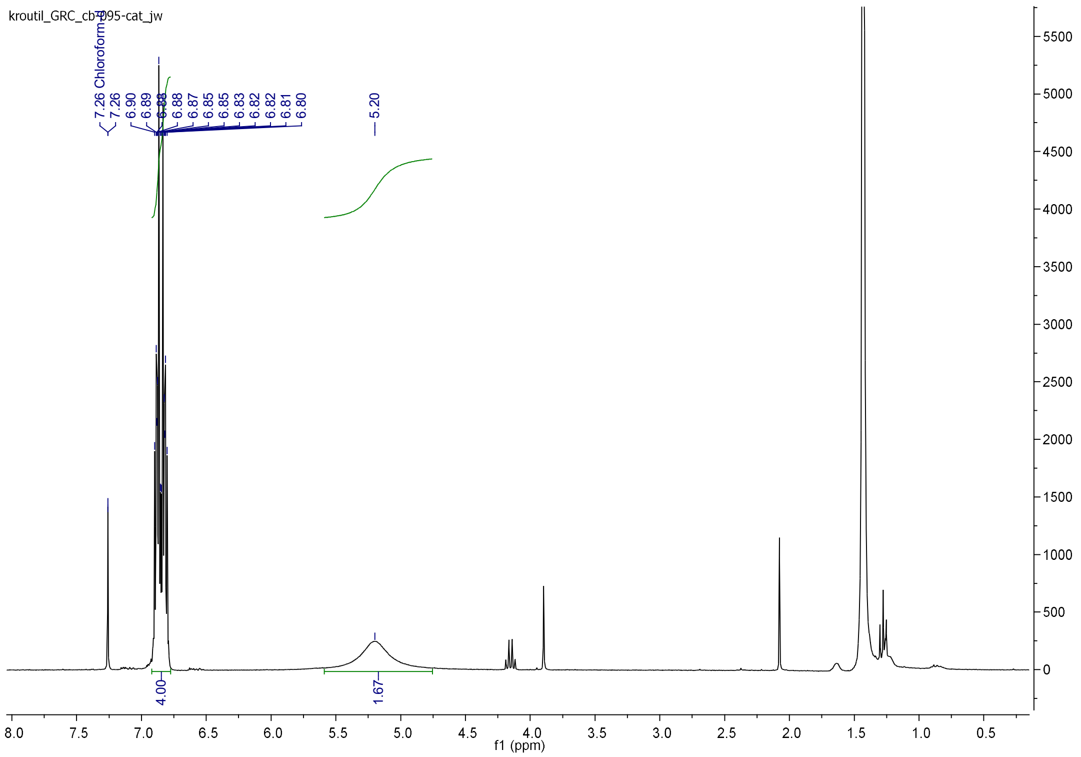

Figure S20. ^{1}H NMR spectra of catechol (**2a**) obtained from biocatalytic reaction after column chromatography (SiO_2). Additional signals refer to the cosolvents ethyl acetate (δ 1.26, δ 2.05, δ 4.12) and cyclohexane (δ 1.43).

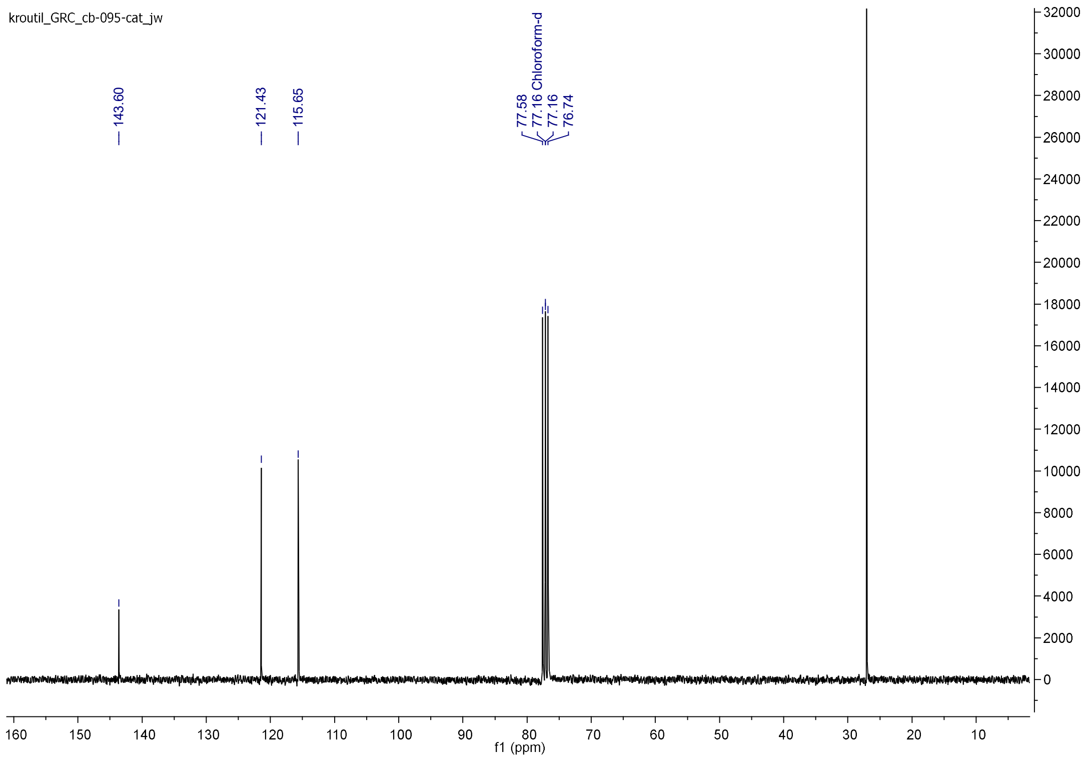

Figure S21. ^{13}C NMR spectra of catechol (**2a**) obtained from biocatalytic reaction after column chromatography (SiO_2). One additional signal refers to the cosolvent cyclohexane (δ 26.94).

Guaiacol (**1a**) isolated column chromatography (SiO_2):

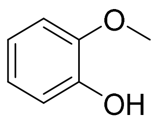

1a

^{1}H NMR (300 MHz, $CDCl_3$) δ 6.97 – 6.83 (m, 4H), 5.63 (s, 1H), 3.89 (s, 3H).
^{13}C NMR (75 MHz, $CDCl_3$) δ 146.7, 145.8, 121.6, 120.3, 114.6, 110.8, 56.0.

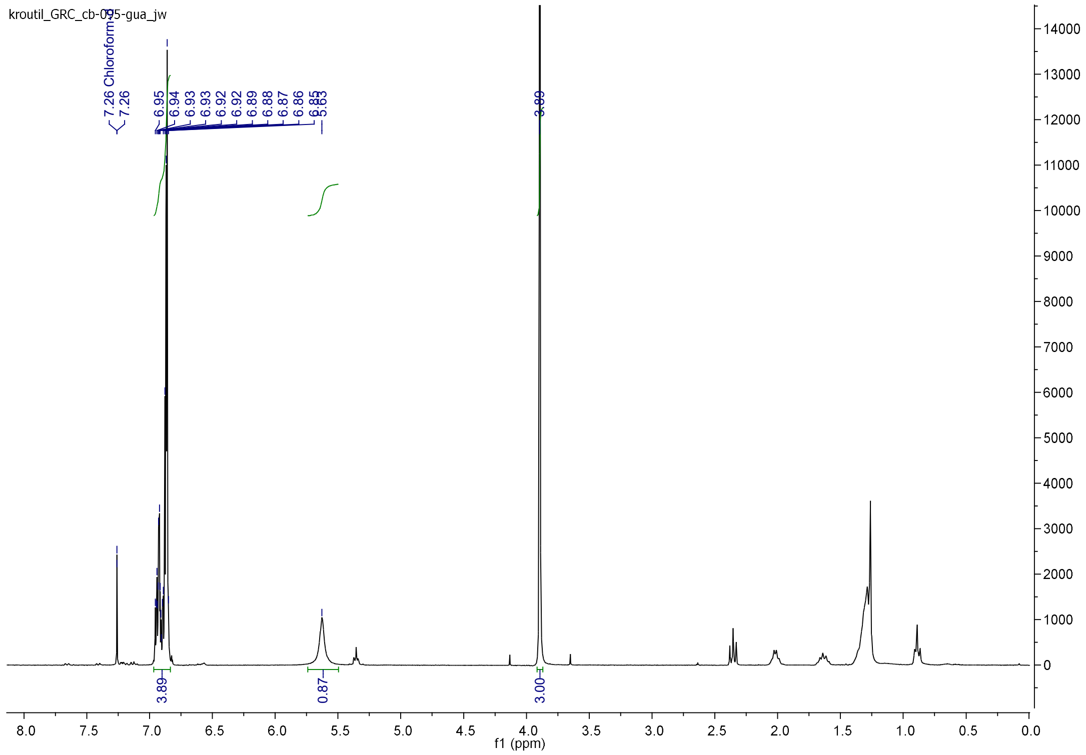

Figure S22. ^{1}H NMR spectra of guaiacol (**1a**) obtained from biocatalytic reaction after column chromatography (SiO_2). Additional signals refer to the cosolvents ethyl acetate (δ 2.05, δ 4.12) and cyclohexane (δ 1.43).

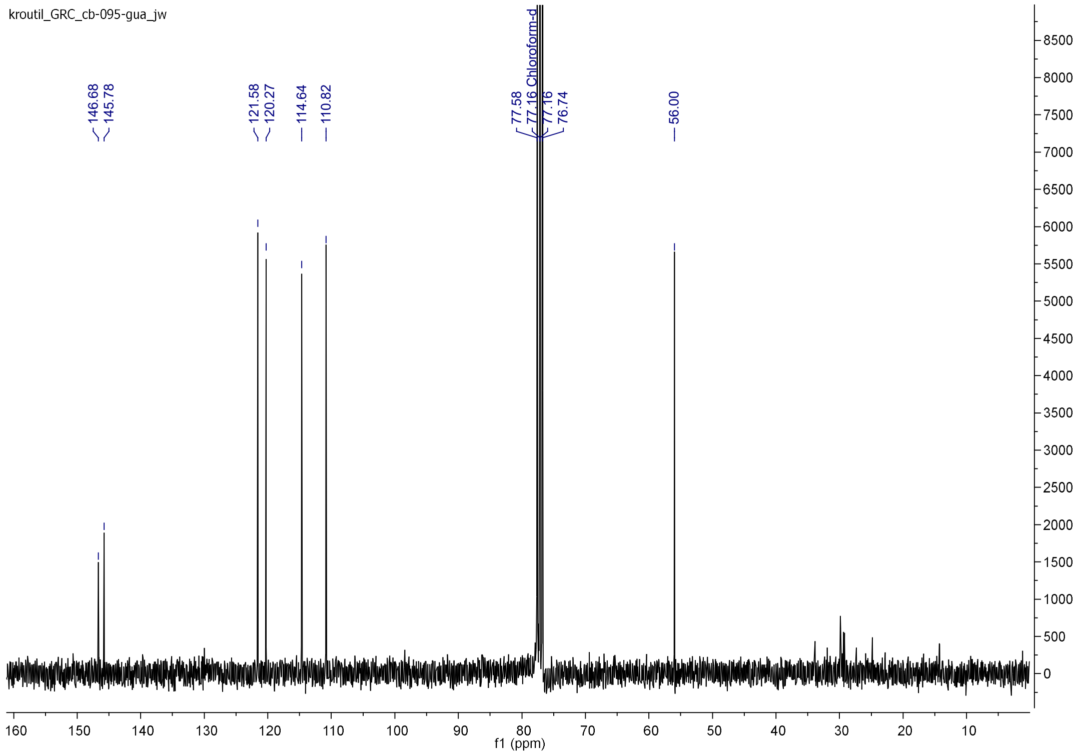

Figure S23. ^{13}C NMR spectra of guaiacol (**1a**) obtained from biocatalytic reaction after column chromatography (SiO_2).

Cis and *trans*-isomers of coniferyl alcohol (**1d**):

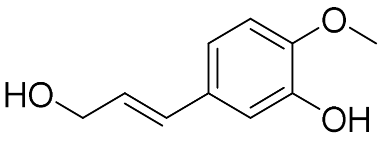

1d

^{1}H NMR (300 MHz, $CDCl_3$) δ 6.85 (m, 6H), 6.52 (m, 2H), 6.28 – 6.16 (m, 2H), 4.33 – 4.27 (m, 4H), 3.90 (s, 3H), 3.89 (s,3H).
^{13}C NMR (75 MHz, $CDCl_3$) δ 146.7, 146.5, 145.8, 145.7, 131.5, 131.1, 130.5, 129.4, 126.9, 126.3, 120.5, 119.2, 114.6, 112.1, 110.7, 108.4, 64.0, 64.0, 56.1, 56.0.

Reference NMR of *trans*-coniferyl alcohol (**1d**):

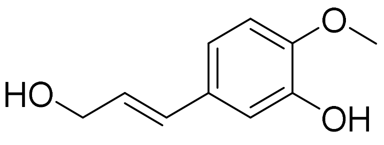

1d

^{1}H NMR (300 MHz, $CDCl_3$) δ 6.89 (m, 3H), 6.53 (d, J = 15.8 Hz, 1H), 6.22 (td, J = 15.8 Hz, 1H), 4.30 (m, 2H), 3.90 (s, 3H).
^{13}C NMR (75 MHz, $CDCl_3$) δ 146.7, 145.7, 131.5, 129.4, 126.3, 120.4, 114.6, 108.4, 64.0, 56.0.

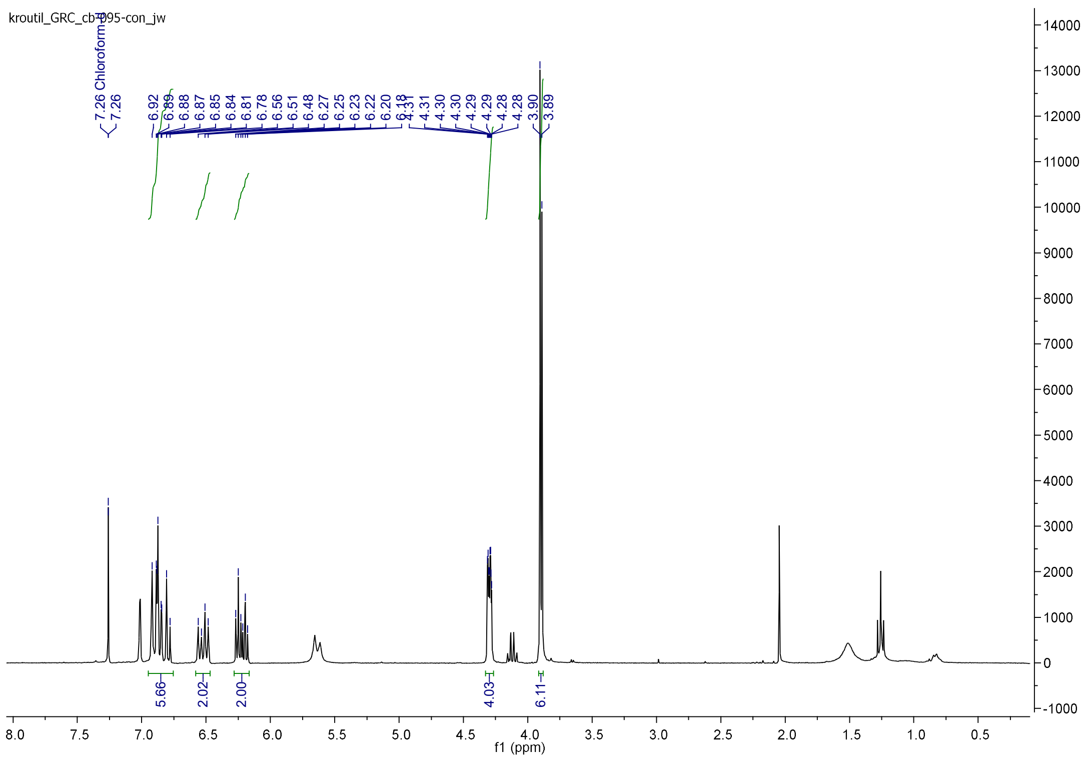

Figure S24. ^{1}H NMR spectra of *cis*- and *trans*-isomers of coniferyl alcohol (**1d**) obtained from biocatalytic reaction after column chromatography (SiO_2). Additional signals refer to the cosolvents ethyl acetate (δ 1.26, δ 2.05, δ 4.12) and cyclohexane (δ 1.43).

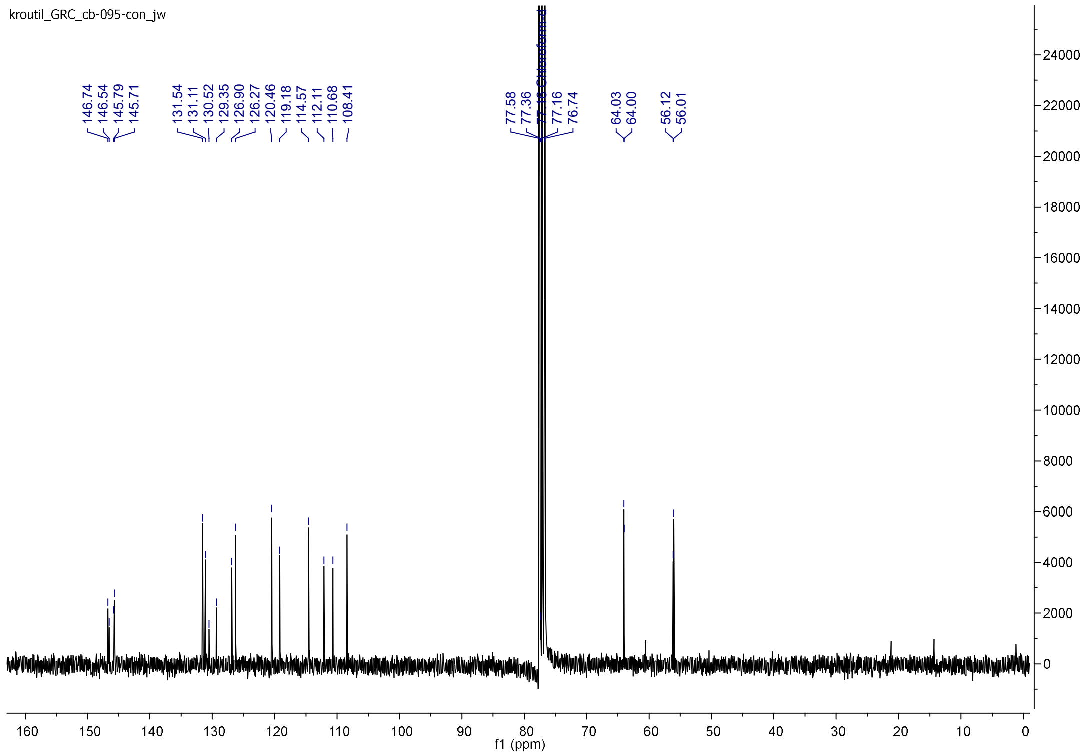

Figure S25. ^{13}C NMR spectra of *cis*- and *trans*-isomers of coniferyl alcohol (**1d**) obtained from biocatalytic reaction after column chromatography (SiO_2). Additional signals refer to the cosolvent ethyl acetate (δ 14.19, δ 21.04, δ 60.49).

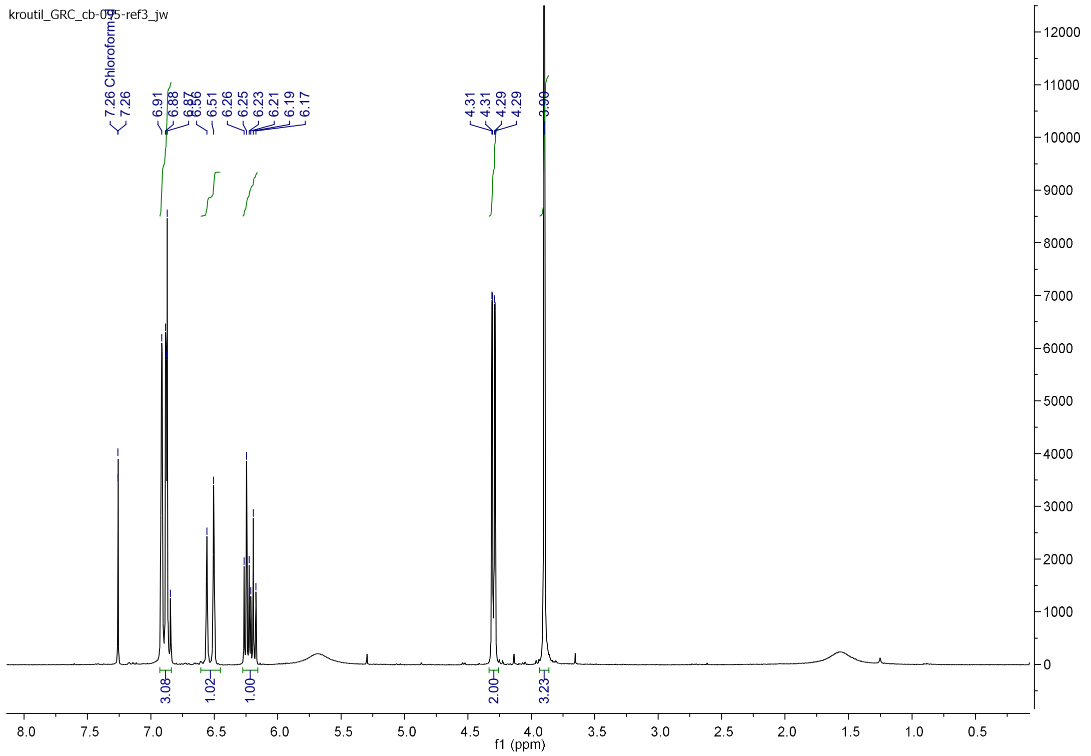

Figure S26. ^{1}H NMR spectra of reference *trans*-coniferyl alcohol (**1d**).

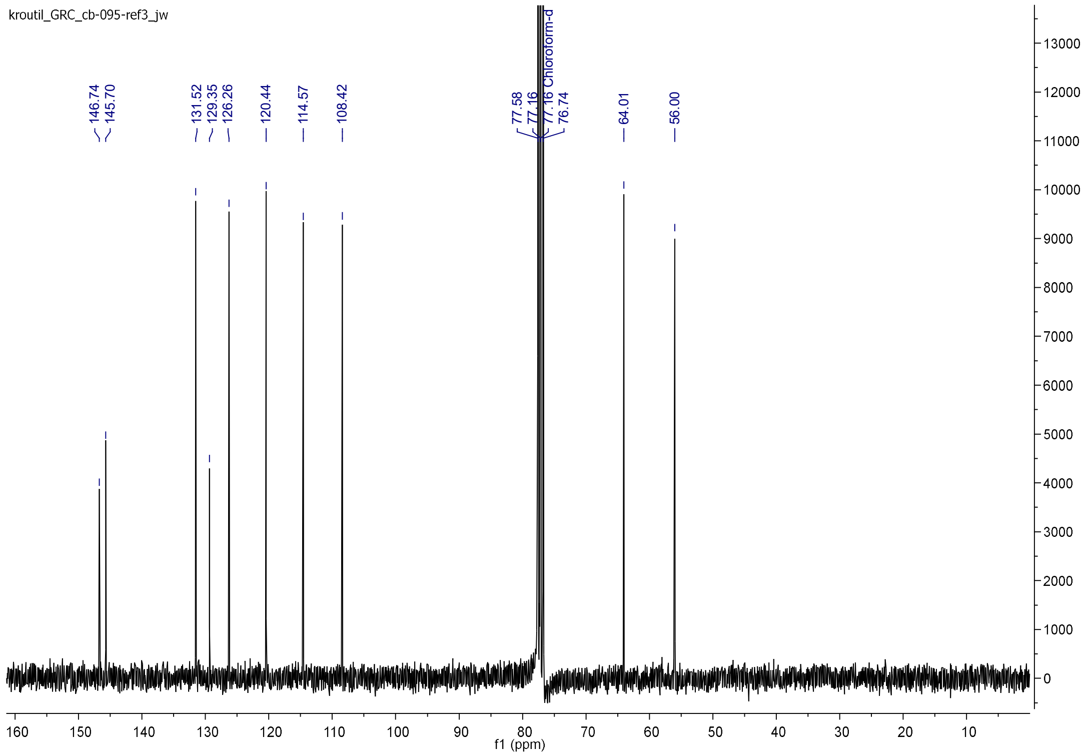

Figure S27. ^{13}C NMR spectra of reference *trans*-coniferyl alcohol (**1d**).

Caffeoyl alcohol (**2d**), NMR data is in accordance with the literature:[6]

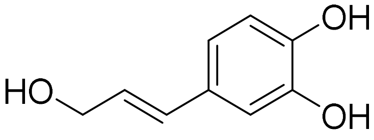

2d

^{1}H NMR (300 MHz, MeOD) δ 6.88 (d, *J* = 1.6 Hz, 1H), 6.75 – 6.67 (m, 2H), 6.43 (d, *J* = 15.8 Hz, 1H), 6.12 (td, *J* = 15.8, 6.0 Hz, 1H), 4.17 (dd, *J* = 6.0, 1.3 Hz, 2H).

^{13}C NMR (75 MHz, MeOD) δ 146.3, 146.3, 132.2, 130.6, 126.6, 119.9, 116.3, 113.9, 63.9.

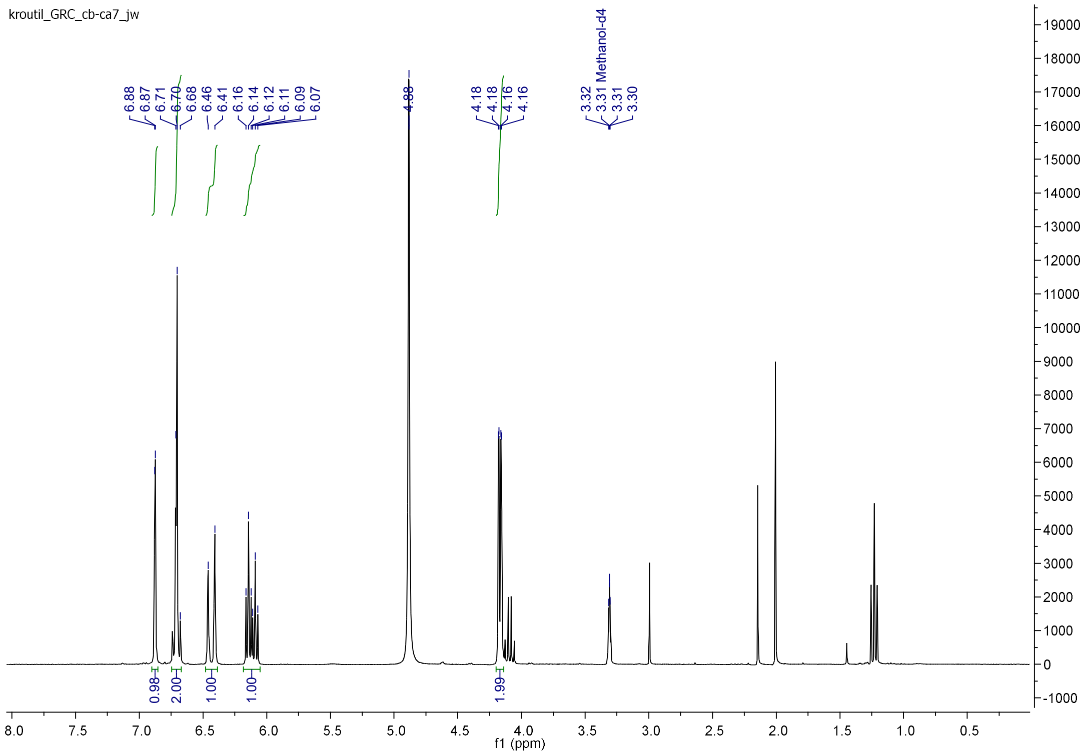

Figure S28. ^{1}H NMR spectra of reaction sample with caffeoyl alcohol (**2d**) isolated column chromatography (SiO_2). Additional signals refer to the cosolvents ethyl acetate (δ 1.24, δ 2.01, δ 4.09) and cyclohexane (δ 1.45).

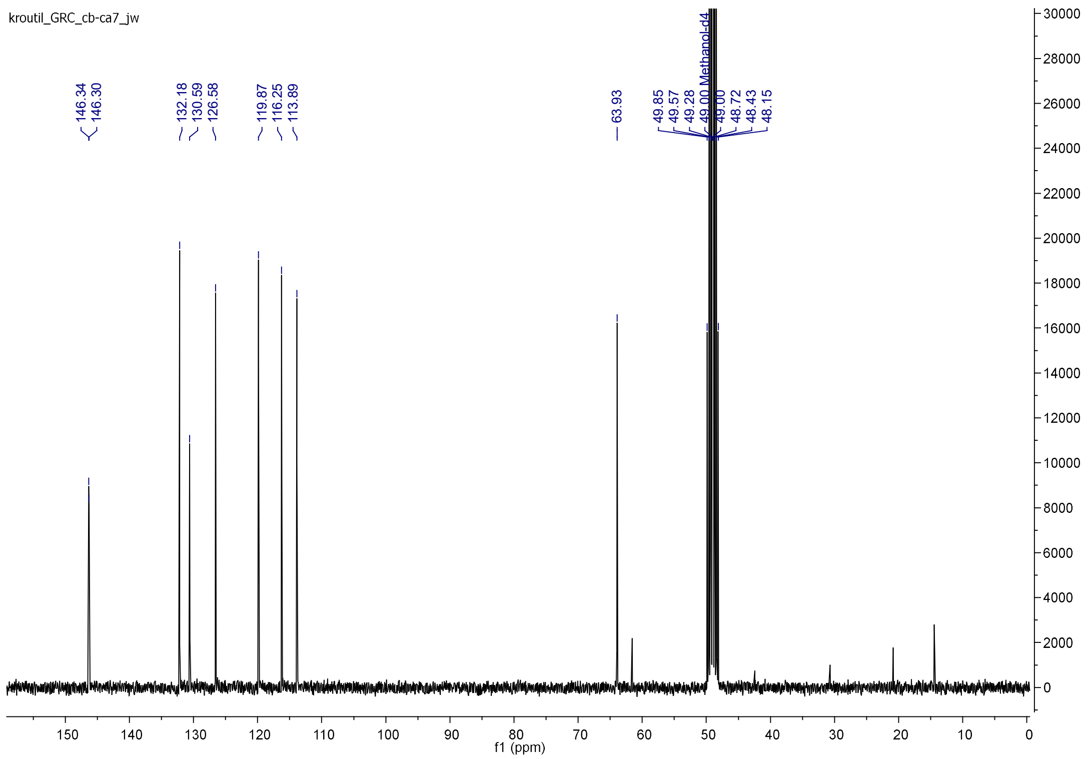

Figure S29. ^{13}C NMR spectra of reaction sample with caffeoyl alcohol (**2d**) isolated column chromatography (SiO_2). Additional signal refers to the cosolvent ethyl acetate (δ 14.49, δ 20.88, δ 61.50).

5. References

(1) Richter, N.; Farnberger, J. E.; Pompei, S.; Grimm, C.; Skibar, W.; Zepeck, F.; Kroutil, W. Biocatalytic Methyl Ether Cleavage: Characterization of the Corrinoid-Dependent Methyl Transfer System from Desulfitobacterium Hafniense. *Adv. Synth. Catal.* **2019**, *361* (11), 2688–2695.

(2) Grimm, C.; Lazzarotto, M.; Pompei, S.; Schichler, J.; Richter, N.; Farnberger, J. E.; Fuchs, M.; Kroutil, W. Oxygen-Free Regioselective Biocatalytic Demethylation of Methyl-Phenyl Ethers via Methyltransfer Employing Veratrol-O-Demethylase. *ACS Catal.* **2020**, *10* (18), 10375–10380.

(3) Farnberger, J. E.; Richter, N.; Hiebler, K.; Bierbaumer, S.; Pickl, M.; Skibar, W.; Zepeck, F.; Kroutil, W. Biocatalytic Methylation and Demethylation via a Shuttle Catalysis Concept Involving Corrinoid Proteins. *Comm. Chem.* **2018**, *1* (1), 1–8.

(4) Richter, N.; Zepeck, F.; Kroutil, W. Cobalamin-Dependent Enzymatic O-, N-, and S-Demethylation. *Trends Biotechnol.* **2015**, *33* (7), 371–373.

(5) Farnberger, J. E.; Hiebler, K.; Bierbaumer, S.; Skibar, W.; Zepeck, F.; Kroutil, W. Cobalamin-Dependent Apparent Intramolecular Methyl Transfer for Biocatalytic Constitutional Isomerization of Catechol Monomethyl Ethers. *ACS Catal.* **2019**, *9* (5), 3900–3905.

(6) Prevost, M. S.; Delarue-Cochin, S.; Marteaux, J.; Colas, C.; van Renterghem, C.; Blondel, A.; Malliavin, T.; Corringer, P.-J.; Joseph, D. Identification of Cinnamic Acid Derivatives As Novel Antagonists of the Prokaryotic Proton-Gated Ion Channel GLIC. *J. Med. Chem.* **2013**, *56* (11), 4619–4630.
